# Whole-genome resequencing-based characterization of a durum wheat landrace showing similarity to ‘Senatore Cappelli’

**DOI:** 10.1371/journal.pone.0291430

**Published:** 2023-09-21

**Authors:** Fernando Tateo, Monica Bononi, Giulia Castorina, Salvatore Antonio Colecchia, Stefano De Benedetti, Gabriella Consonni, Filippo Geuna

**Affiliations:** 1 Department of Agricultural and Environmental Sciences – Production, Landscape, Agroenergy (DISAA), University of Milan, Milan, Italy; 2 Council for Agricultural Research and Economics, Research Center for Cereal and Industrial Crops (CREA-CI), Foggia, Italy; 3 Department of Food, Environmental and Nutritional Sciences, University of Milan, Milan, Italy; National Bureau of Plant Genetic Resources, INDIA

## Abstract

Durum wheat (*Triticum turgidum* spp. *durum*) is a major cereal adopted since antiquity to feed humans. Due to its use, dating back several millennia, this species features a wide genetic diversity and landraces are considered important repositories of gene pools which constitute invaluable tools for breeders. The aim of this work is to provide a first characterization of a wheat landrace, referred to as ‘TB2018’, that was collected in the Apulia region (Southern Italy). ‘TB2018’ revealed, through visual inspection, characters reminiscent of the traditional variety ‘Senatore Cappelli’, while exhibiting a distinctive trait, i.e., reduced stature. Indeed, the comparison with a set of Italian durum wheat cultivars conducted in this study, in which 24 CPVO plant descriptors were adopted, placed the ‘TB2018’ landrace in proximity to the ‘Senatore Cappelli’ cultivar. In addition, the close similarity between the two genotypes was confirmed by the analysis of the seed protein pattern. A relative reduction was detected for ‘TB2018’ root elongation in the early stages of plant growth. The ‘TB2018’ genome sequence, obtained through low-coverage resequencing and comparison to the reference ‘Svevo’ cultivar is also reported in this study, followed by a genome-wide comparison against 259 durum wheat accessions that placed ‘TB2018’ close to the ‘Cappelli’ reference. Hundreds of genes putatively affected by variants that possess Gene Ontology descriptors were detected, among which some were shown to be putatively linked to the morphological traits that distinguish ’TB2018’ from ’Senatore Cappelli’, Overall, this study poses the basis for a possible exploitation of ’TB2018’ *per se* in cultivation or as a source of alternative alleles in the breeding of traditional cultivars. This work also presents a genomic methodology that exploits the information contained in a low-depth, whole-genome sequence to derive genotypic data useful for cross-platform (chip data) comparisons.

## Introduction

Durum wheat (*Triticum turgidum* subsp. *durum* (Desf.) Husn.) is the most important food crop widely grown across the Mediterranean basin. In the European Union (EU) it is grown on over 2.4 × 10^6^ ha, which produce 7.9 × 10^6^ Mg of grain with an average yield of 3.3 Mg ha^−1^. Italy is the leading producer with 4.2 × 10^6^ Mg of grain obtained on 1.3 × 10^6^ ha with an average yield of 3.2 Mg ha^−1^ [1]. Italy also represents the most important EU producer country of organic durum wheat with a cropping area constantly increasing in recent years, and reaching about 87.8 × 10^3^ ha, providing 292 × 10^3^ Mg of grain [2]. Regions of southern Italy provide about 70% of this durum wheat production used mainly for pasta, and also for bread making.

A little more than two years following the Italian national obligation to indicate the origin of wheat on pasta labels, the value of durum wheat in Italy has grown by 20% [3]. This is due to the boom of 100% Italian wheat pasta along with the collapse of imports from Canada due to concerns relating to the use of glyphosate in pre-harvest according to methods prohibited in Italy. This trend has caused the collapse of durum wheat sowing in Canada, where farmers have decided to cultivate 18.8% less durum wheat land than previous year.

The phenomenon of the return to local varieties (with cv. ‘Senatore Cappelli’ as the leader) has exploded in Italy in recent years, with a growth trend that in the 2017–2018 campaign quintupled the cultivated areas, going from 1,000 hectares in 2017 to 5,000 currently [3].

Southern Italy is one of the regions where, historically, cereal crops were most cultivated and where the durum wheat varietal biodiversity is particularly high [5]. Old and new durum wheat commercial varieties are currently cultivated, but so are many ancient landraces or populations characterized by specific bio-morphological traits and qualitative features [4, 5].

In recent years, all over the world, the agri-food sector is paying renewed attention to local and traditional productions. The major drivers of this shift are a response to the lack of diversification due to globalization and social and economic changes, but also an increased attention to nutritional and health aspects of food. In Italy too, this trend has led to the rediscovery and reuse of landraces of both wheat and other crops, addressing the needs for an increasingly demanding market. The steadily rising prices of these local productions are attracting the producers’ interest, often turning an unprofitable activity into a renewed professional opportunity also for young entrepreneurs. Furthermore, many recent research studies confirm the high nutraceutical and ecological value of landraces, both for their high content of antioxidant compounds and their natural aptitude for organic production [6]. The active field of recovery of the precious genetic resources hidden in natural landraces is evidenced by a diverse array of methods developed to investigate and identify naturally occurring variants [7, 8]. In this respect, it is of no surprise that several huge research initiatives have undertaken the characterization of the phenotypic, genetic, and genomic diversity of wide collections of landraces [9, 10].

This growing interest in local landraces has also inspired a search of effective and objective identification methods, able to distinguish landraces [11]. One of the most valuable and reliable approaches is provided by the modern genome sequencing technologies. With the costs of resequencing and genotyping steadily falling, increasing amounts of variation data are being produced and stored in databases. To effectively annotate this huge amount of data, access to considerable computational resources and genomic annotation databases are nowadays a prerequisite. Often, the most valuable information about a variant is its alleged effect on the affected transcript(s) of the relative gene. This may aid selection of variations for genotyping studies and, in turn, be a determinant in the discovery of target loci and their biological role.

The set of experiments described in this work were performed in the framework of an investigation of landraces undergoing genetic erosion to determine agronomic attitude and qualities.

The aim of this work is to provide a first characterization of a durum wheat landrace, referred to as ‘TB2018’, which was found in the Lecce province, Italy, and disclosed, through visual inspection of plant and seed traits, similarity with the historical and celebrated ‘Senatore Cappelli’ cultivar. This registered variety is characterized by wide adaptability, a greater number of kernels per ear and an excellent quality of the flour obtained [12]. ‘TB2018’ was also selected since it exhibited reduced plant stature, a trait of agronomic relevance that attracted our interest.

The morpho-phenological, agronomic and seed quality tests performed in this study provide the most accurate and thorough description of a selected landrace and sustain the hypothesis that the two accessions are related. Beside confirming the observed difference in plant elongation, the present work has highlighted an additional distinct trait related to root elongation in the early phase of development.

Over the past few decades, global wheat yield has been significantly increased from just over 1 tonne per hectare in the early 1960s to around 3.5 tonnes in the past decade. However, it has been estimated that genetic gain has been diminished in improved cereals, including wheat, and that only 10% of the natural diversity has been captured in the elite germplasm of our major crops [9]. If we consider that over the coming years, wheat production will be challenged by a progressively mutable climate, this genetic loss needs to be urgently counterbalanced by expanding the allelic diversity available for breeding programs. In this context, the contribution of this study consists in an in-depth characterization of a novel variety characterized for distinctive traits of agronomic interest. By exploiting the recent information produced by the sequence of the first durum wheat genome [13], our study provides through low-coverage resequencing, a first glimpse of the ‘TB2018’ landrace genome identity. To our knowledge, this represents the first genomic sequence available for a local Italian durum wheat variety, in which novel genetic variants will be accessible for future research studies as well as for being adopted in breeding programs.

## Materials and methods

### Plant materials

Seeds of ‘TB2018’ were derived from about 50 almost ripe ears of wheat found by chance in a private property located in the territory of Matino (Lecce province, Apulia region, Southern Italy), on a private property road, and at the following coordinates: 40° 00’N, 18° 11’ E, 75 m a.s.l. In living memory, there is no evidence that wheat was produced on this land. After a year of growth in the same place, the seeds were sown in an experimental field at the CREA Research Center for Cereal and Industrial Crops (CREA-CI), S.S. 673, km 25,200, 71122 Foggia, Apulia region, Italy.

*Triticum turgidum* ssp. *durum* cultivars ‘Marco Aurelio’ and ‘Senatore Cappelli’ were obtained from “Società Italiana Sementi” (SIS, San Lazzaro di Savena (Bologna), Italy, www.sisonweb.com).

The pedigree/genealogy of ‘Marco Aurelio’ is ‘Orobel’//’Arcobaleno’/’Svevo’. ‘Senatore Cappelli’ is an old cultivar of durum wheat, dating back to 1915, obtained by the geneticist Nazareno Strampelli at the Research Center for Cereal Growing in Foggia, Italy, through genealogical selection (nr. 231/1915) carried out in Foggia on the North African population ‘Jenah Rhetifah’.

The set of Italian durum wheat cultivars used in the morpho-physiological characterization is provided in S1 Table.

### Plant growth conditions

A quality testing experiment, programmed for a two-year period, was started in autumn 2018 at the CREA (Consiglio per la Ricerca in Agricoltura e l’analisi dell’Economia Agraria) Research center for cereals and industrial crops (Foggia, Italy; 41° 27′ 36” N, 15° 30′ 05” E; 75 m a.s.l.) on clayey soil (Typic Chromoxerert). The main characteristics of the soil were 30% clay, 25% sand; pH 7.5; 12.5 g kg^-1^ total C.

The climate is typical of a Mediterranean environment characterized by the presence of a dry season between May and September. Based on 62 years of climatic data (starting 1955), the annual mean temperature is 15.7°C (range 9.7–21.8°C), with a precipitation of 529 mm, and with a high variability, especially for rainfall (range 272–803 mm).

The experiment was established with 11 wheat accessions and 2 fertilization levels in a randomized block design with 2 replicates and elementary plots of 10 m^2^, repeated for two years for a total of 44 plots per year. Within this experiment, we set an *a priori* contrast between the main effect on ‘TB2018’ and ‘Senatore Cappelli’ for the analyzed variable.

Normality of residual distribution and homogeneity of variances was checked, respectively, with the Shapiro-Wilk and the Levene tests [14]. The sowing was carried out with a plot seeder on 7 December 2018. The germinability was 95% and 350 germinable seeds were distributed per m^2^. In pre-sowing, 92 kg/ha^-1^ of phosphorus (as P_2_O_5_) were distributed; in the growing period, three fractional fertilizations were carried out with 120 kg/ha^-1^ of nitrogen (ammonium nitrate at 26%): i) tillering (BBCH 24) with 60 kg/ha^-1^ (15 March 2019), ii) lifting II node (BBCH 32) with 30 kg/ha^-1^ (05 April 2019) and iii) barrel (BBCH 41) with the last 30 kg/ha^-1^ (23 April 2019).

The control of wild herbs/weeds was carried out with a treatment (herbicide) based on an Atlantis + Biopower + Zenit formulation. The earing date was about 34 days from 1 April and the average height was 110 cm.

The harvest was carried out at the physiological maturation stage corresponding to grade 92 of the Zadoks scale [15], with a plot combine. The average yield was 4.11 t/ha.

### Morpho-physiological analysis

For the morphological analysis the descriptors employed were those described in the CPVO TP/120/2 protocol for Distinctness, Uniformity and Stability (DUS) [16, 17] and the corresponding UPOV web site [18]. The growth stages were referred to by the decimal codes of [15]. A principal component analysis (PCA), multivariate approach was followed employing the above-mentioned descriptors, to describe and classify the accession under study. A set of Southern Italian durum wheat cultivars and the corresponding descriptors available online, following the same coding scheme, were chosen as reference. The numerical values corresponding to indicators listed in the CPVO/UPOV identification protocols were used to compose a spreadsheet file (S1 Table) that was used as input for the R-based software ‘prcomp’ [19]. The resulting PCA data object was plotted using the R-based software ‘ggbiplot’ v.0.55 [20].

### Total protein extraction and SDS-PAGE analysis

Whole mature and dry seeds were ground to a meal and flours were suspended in an extraction buffer (Urea 6 M, SDS 2%, DTT 5 mM; 1:10 w/v) and stirred for 2 hours at room temperature. The suspension was then centrifuged at 10,000 x g at 4°C for 30 min to separate solubilized proteins. Protein quantification was performed with the Bradford method [21].

SDS-PAGE analysis was carried out according to [22] on 12% polyacrylamide gel under reducing conditions. Gels were stained with Coomassie Blue G-250 (Bio-Rad, Hercules, CA, USA) and the relative molecular mass of polypeptides was determined by comparison with standard proteins (GE Healthcare, Chicago, IL, USA) [23].

### *In vitro* growth conditions and phenotypic characterization

Seeds were sterilized by soaking in a solution of 50% bleach and sterile water for 15 minutes followed by a couple of quick washes in sterile water and then five washes for 5 minutes each in sterile water. Seeds were germinated and grown in 50 mm diameter plates at 22°C for 3 days in the dark on 3M filter paper soaked in sterile water or on an alternative substrate composed of inert vegetable fiber soaked in sterile water.

For the phenotypic observations, images of seedlings were taken. The total root and lateral root number were counted. The longest primary seminal root length and the lateral root length of each seedling were also measured using the ImageJ software [24].

### Genomic DNA extraction, library preparation and sequencing

Total genomic DNA was extracted from 10 g of ground seeds using the Qiagen Plant DNA kit (Qiagen). DNA quality was assessed by conventional 0.8% (w/v) agarose gel electrophoresis [25] and spectrophotometry using a Nanodrop (ThermoFisher) [26].

The Celero TM DNA-Seq kit (NuGEN, San Carlos, CA, USA) was used for library preparation following the manufacturer’s instructions. Both input and final library were quantified by a Qubit 2.0 Fluorometer (Invitrogen, Carlsbad, CA, USA) and quality tested by an Agilent 2100 Bioanalyzer High-Sensitivity DNA assay (Agilent Technologies, Santa Clara, CA, USA). Libraries were then prepared and sequenced on a NovaSeq 6000 (Illumina) instrument in paired-end 150 mode (IGATech, Udine, Italy).

### Bioinformatic analysis of the ‘TB2018’ genome sequence

A primary bioinformatic analysis included the following steps:

- Base calling and demultiplexing. Processing raw data for both format conversion and de-multiplexing by Bcl2Fastq v.2.0.2 of the Illumina pipeline [27].
- Adapter sequences were masked with Cutadapt v1.11 [28].

A low (5X) coverage of the ‘TB2018’ genome, yielding 552.82 million reads, was estimated sufficient for the scope of the analysis.

Quality control and mapping of reads against the reference genome of *Triticum turgidum* L. var. *durum* cv. ‘Svevo’ (genome sequence: GCA_900231445.1.fasta; [13]) was done through the NGSEP (v.4.01) software [29]. For mapping reads an index of the ‘Svevo’ reference genome was first obtained through the Bowtie v.2.4.3 software [30].

Quality metrics and statistics of reads were obtained through the Picard v.2.26.3 software [31].

The identification of sequence variants (SNPs, InDels) was performed through the NGSEP software using the VCFannotate command of the NGSEP (v.4.01) software [29]. Ploidy was addressed using the appropriate flags as described in the NGSEP manual [29, 32].

For the annotation to the *Triticum durum* reference genome, the GFF3 file version 1.51 was used [33]. For all the analyses the high-performance computing (HPC) cluster at the University of Milano [34] was employed running under a CentOS 7 operating system with a minimum of 16 cores and 64 GB RAM for the most demanding runs of index building and read mapping.

The effect of any structural and single nucleotide variant detected by the comparison of the ‘TB2018’ genome against the ‘Svevo’ reference genome was estimated through the Variant Effect Predictor (VEP) v.104 software [35, 36] hosted at the Ensembl web site [37]. The list of VEP parameters and values used for the analysis is provided (S2 Table).

Variants were classified according to the VEP scheme [38], and further crossed with biological descriptors of gene function retrieved through the Gene Ontology [39] and the BioMart [40] online bioinformatic resources. The output files following VEP analysis at the single chromosome level were used to filter genes whose mutation(s) are expected to produce a relevant effect on protein coding (“IMPACT = HIGH” flag).

The analysis of repetitive elements was done on the GenSAS server [41] using the RepeatMasker v.4.1.1 program [42] that screens DNA sequences for interspersed repeats and low complexity DNA sequences. The output of the program is a detailed annotation of the repeats that are present in the query sequence. The details of parameters followed is reported (S1 Text).

The results of the repetitive element analysis are made available through the GenSAS server via the Apollo [43] genomic annotation platform which is coupled with JBrowse [44] for viewing feature alignments and for manual curation. Raw output data in GFF3 format can be accessed (https://doi.org/10.13130/RD_UNIMI/SLKJLY).

### Genome-wide comparison of ‘TB2018’ against durum wheat germplasm

The methodology followed was based on the derivation, from the whole-genome resequencing data of ‘TB2018’, of genotypes at selected SNP loci to compare with a collection of 259 durum wheat cultivars already genotyped for the same loci [45]. The whole-genome sequence of ‘TB2018’ described in the above section was used to derive the SNP positions corresponding to the 3,541 SNP loci selected in [45].

The list of 3,541 probes spanning the SNP loci was first provided (Dr. Francesca Taranto, personal communication) and represents a subset of the probes of the Illumina iSelect 15k wheat SNP array aligned to ‘Durum’ and ‘Zavitan’ genomes. The chip contains 13,600 gene-associated SNP markers [46] and is an optimized and reduced version of the 90k iSELECT SNP-chip described by [47].

The iSelect 15k array chip sequences for both Durum and Zavitan wheat species are now available on the web page of the GrainGenes Database of Triticeae and Avena [48].

The following analytical pipeline was followed:

1. the list of probes was used to run a multiple BLAST v. 2.13.0+ [49] search against a local database generated from the ‘TB2018’ resequenced consensus genome with the parameters listed in S1 Text.
2. the output of the BLAST search was formatted to correct the positions of the SNPs relative to the coordinates of the ‘TB2018’ resequenced genome. Such coordinates were used as an input file in the next step to obtain the corresponding genotypes through the ‘getfasta’ command of the ‘Bedtools’ v. 2.25.0 software [50].
3. the output of ‘bedtools getfasta’ was used to derive the complementary sequence at each SNP position and the list of separate SNPs was joined to form a continuous nucleotide string (FASTA pseudomolecule) that was used for comparison to the corresponding sequences of the 259 reference wheat cultivars of [45];
4. the multiple sequence alignment and the phylogenetic tree were generated using the ClustalW-based algorithm and pipeline provided by the EMBL-EBI facility [51].

For sake of reproducibility of the analysis and comparison with data shown in [45] a neighbor-joining tree was also generated using MEGA v.11 [52] and a total of 1,000 replicates were used to generate bootstrap values. The FigTree v.1.4.4 software [53] was used for the graphical visualization of the tree.

To further investigate the placing of ‘TB2018’ against the five “Cappelli” entries from [45], the G-DIRT software [54] was run using the set of 3,509 SNP loci retained from the above analysis and formatted according to the instructions (S2 Text) with the parameters shown in S3 Text.

## Results

The wheat landrace ‘TB2018’, found in the Lecce province (Apulia region, Italy), revealed, through visual inspection of its plant architecture and seed morphology, similarity with the durum wheat registered variety ‘Senatore Cappelli’ (S1 Fig). Visual analysis also evidenced a distinctive trait consisting of a reduction in plant stature.

A characterization of ‘TB2018’ for agronomic, morphological, and biochemical parameters, and the comparison to the registered ‘Senatore Cappelli’ cultivar have been performed in this study. In addition, to strengthen the (preliminary) characterization of this landrace, a whole-genome resequencing was performed, and the obtained genome sequence was compared with the *Triticum turgidum* spp. *durum* reference genome. Moreover, a novel genomic pipeline was set up to compare the whole-genome resequencing data of ‘TB2018’ with the chip-based genotypes of a set of 259 durum wheat cultivars and infer the phylogenetic relationships among them.

### Agronomic parameters

**Fig 1:**
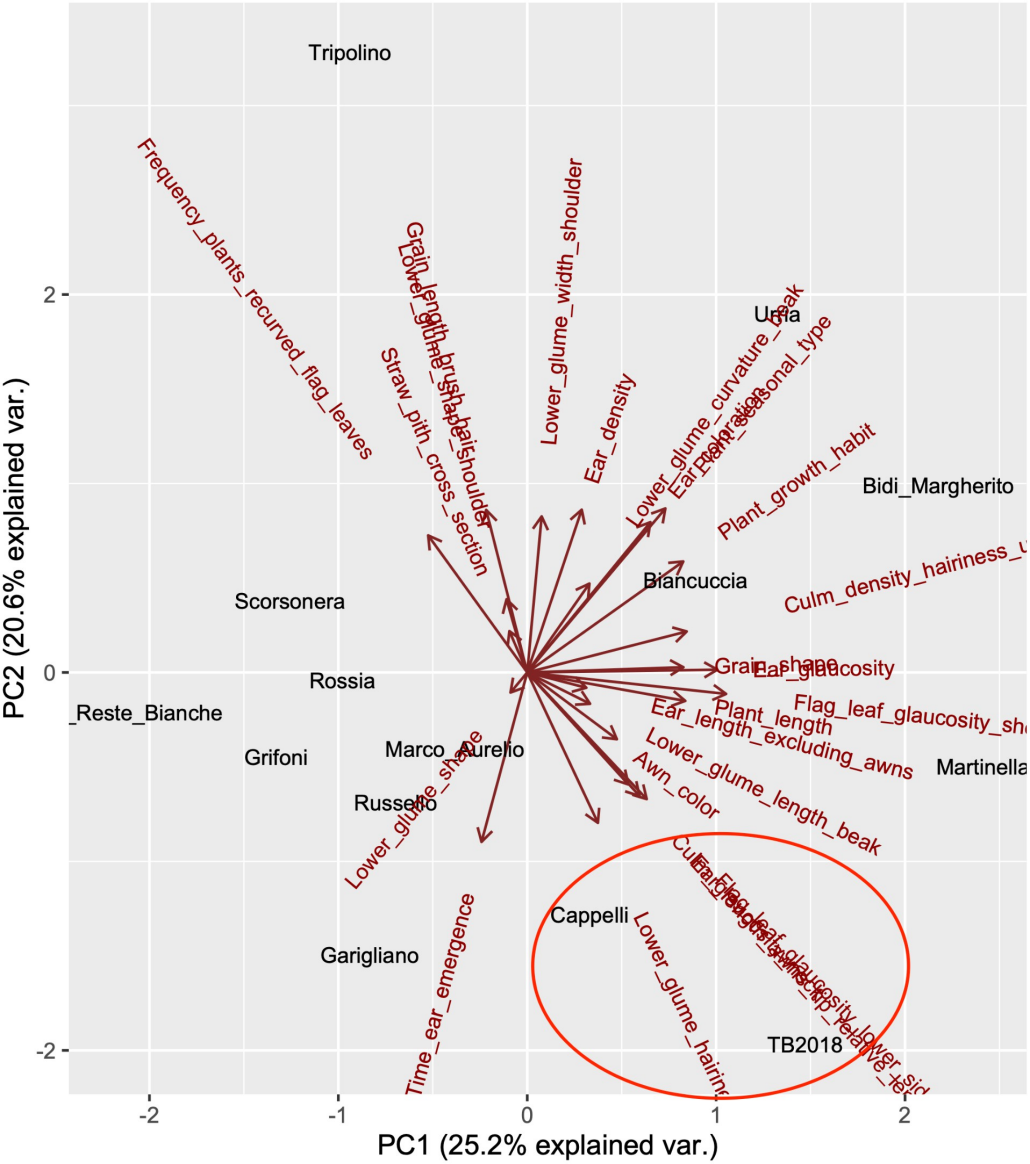
Principal Component Analysis (PCA) of durum wheat accessions based on morphological descriptors. The first two components are shown in the X and Y axes, respectively. Arrows show how each descriptor contributes to the two principal components. The red oval highlights the proximity of ‘TB2018’ to ‘Senatore Cappelli’.

The ‘TB2018’ landrace was included in an agronomic field trial for a set of reference Southern Italian durum wheat cultivars, in which, as part of the morpho-physiological characterization, a principal component analysis investigation was performed, considering 24 CPVO plant descriptors encompassing the major morphological descriptors of the species. The results show the relative contribution of each morphological trait (arrows in Fig 1) in the separation of the analyzed cultivars in the first two principal components, contributing to 25.2% and 20.6% of the total variance, respectively.

Interestingly, the analysis placed the ‘TB2018’ landrace in proximity to the ‘Senatore Cappelli’ cultivar (oval in Fig 1), further supporting the hypothesis that the two are somehow related.

The comparison of ‘Senatore Cappelli’ and ‘TB2018’, was achieved by cultivation under standard agronomic conditions. The Shapiro-Wilk and the Levene tests excluded a significant deviation from normality and homogeneity of variances. No interaction between year, fertilization level and accessions resulted significant. The main effect of accession resulted significant (p< 0.01) as well as the contrast between the two accessions. Data analysis confirmed the striking evidence that ‘TB2018’ featured a shorter stem (average 110 cm) relative to ‘Senatore Cappelli’ (average 135 cm) which could provide an increased resistance to lodging.

### Seed protein analysis confirms the similarity between ‘TB2018’ and ‘Senatore Cappelli’

A comparison between ‘TB2018’ and ‘Senatore Cappelli’ was performed through the analysis of soluble proteins extracted from flour obtained from mature kernels. The two cultivars were also compared to the ‘Marco Aurelio’ cv. used as control. Proteins extracted from ‘TB2018’ and ‘Senatore Cappelli’ had comparable yields of 19.3 ± 0.4 and 21.2 ± 0.5 mg of proteins/g of meal, while in ‘Marco Aurelio’ a slightly higher protein solubilization was obtained, yielding up to 26.6 ± 0.9 mg of proteins/g of meal.

**Fig 2:**
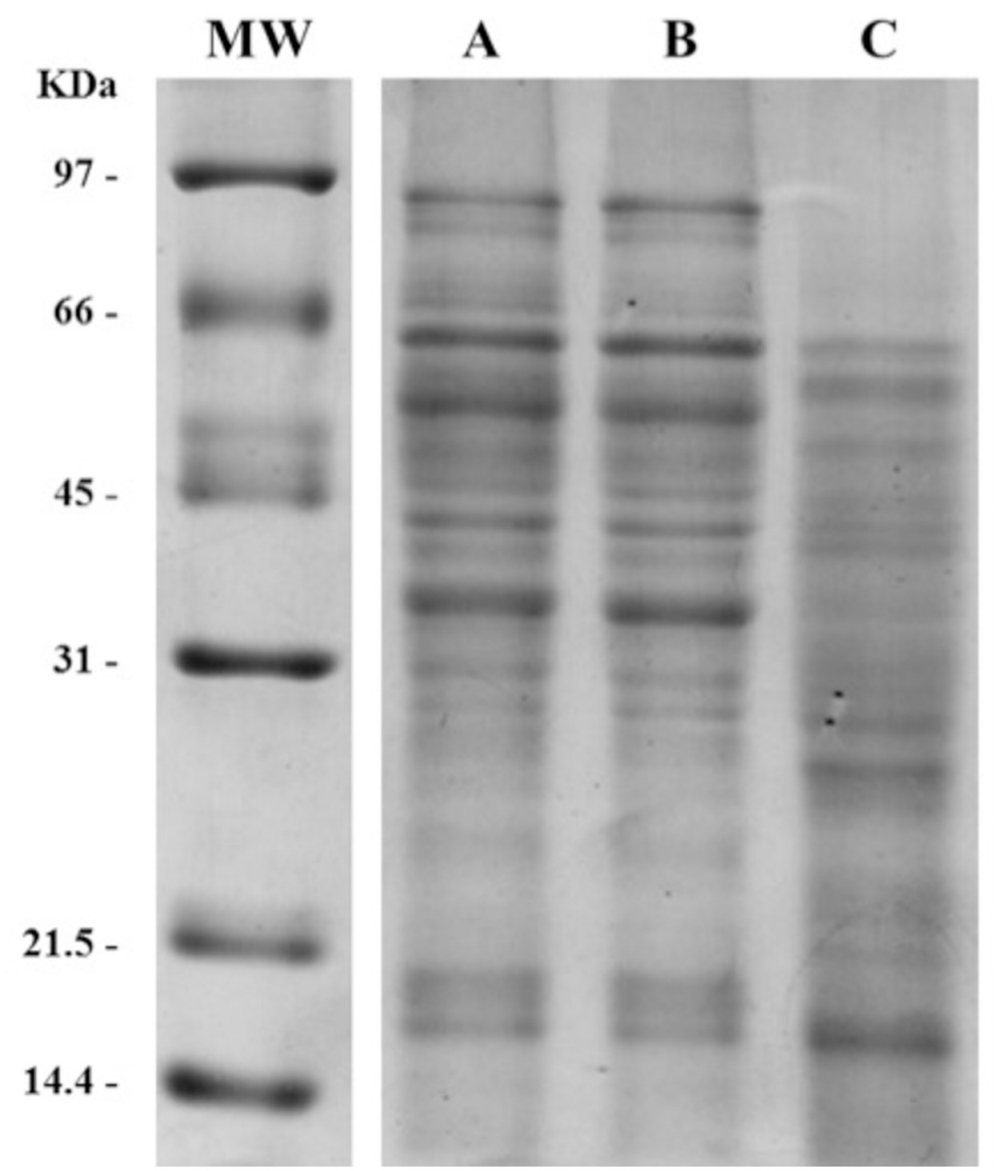
SDS-PAGE analysis of total protein extracts from seeds of the three durum wheat cultivars examined, performed under reducing conditions. A: ‘TB2018’; B: ‘Senatore Cappelli’; C: ‘Marco Aurelio’; MW: molecular weight standard.

The electrophoretic separation performed under reducing conditions allowed the qualitative comparison of total proteins among the three cultivars. Protein distribution patterns of ‘Senatore Cappelli’ (Fig 2 lane A) and ‘TB2018’ (Fig 2 lane B) displayed high similarity, while both patterns clearly differed from that of ‘Marco Aurelio’(Fig 2 lane C).

‘Senatore Cappelli’ and ‘TB2018’ presented a higher content of proteins with molecular weights ranging from about 100 to 65 kDa, among which High-Molecular Weight (HMW) glutenins can be found (Fig 2 lanes A and B). These samples also differed from ‘Marco Aurelio’ for the electrophoretic pattern of proteins between 60 and 30 kDa where gliadins can be found [55, 56]. In addition, the Marco Aurelio pattern reported in lane C showed bands in the molecular range of about 30–21 kDa that could belong to Low-Molecular Weight (LMW) glutenins, while lanes A and B presented a lower content of these proteins.

### Analysis of early developmental stages of plant growth revealed distinct root traits for the ‘TB2018’ landrace

To analyze their seedling phenotype, seeds of ‘TB2018’, ‘Senatore Cappelli’ and ‘Marco Aurelio’ were germinated in controlled conditions. After three days, germination rate and shoot elongation did not reveal any significant difference among the three accessions.

**Fig 3:**
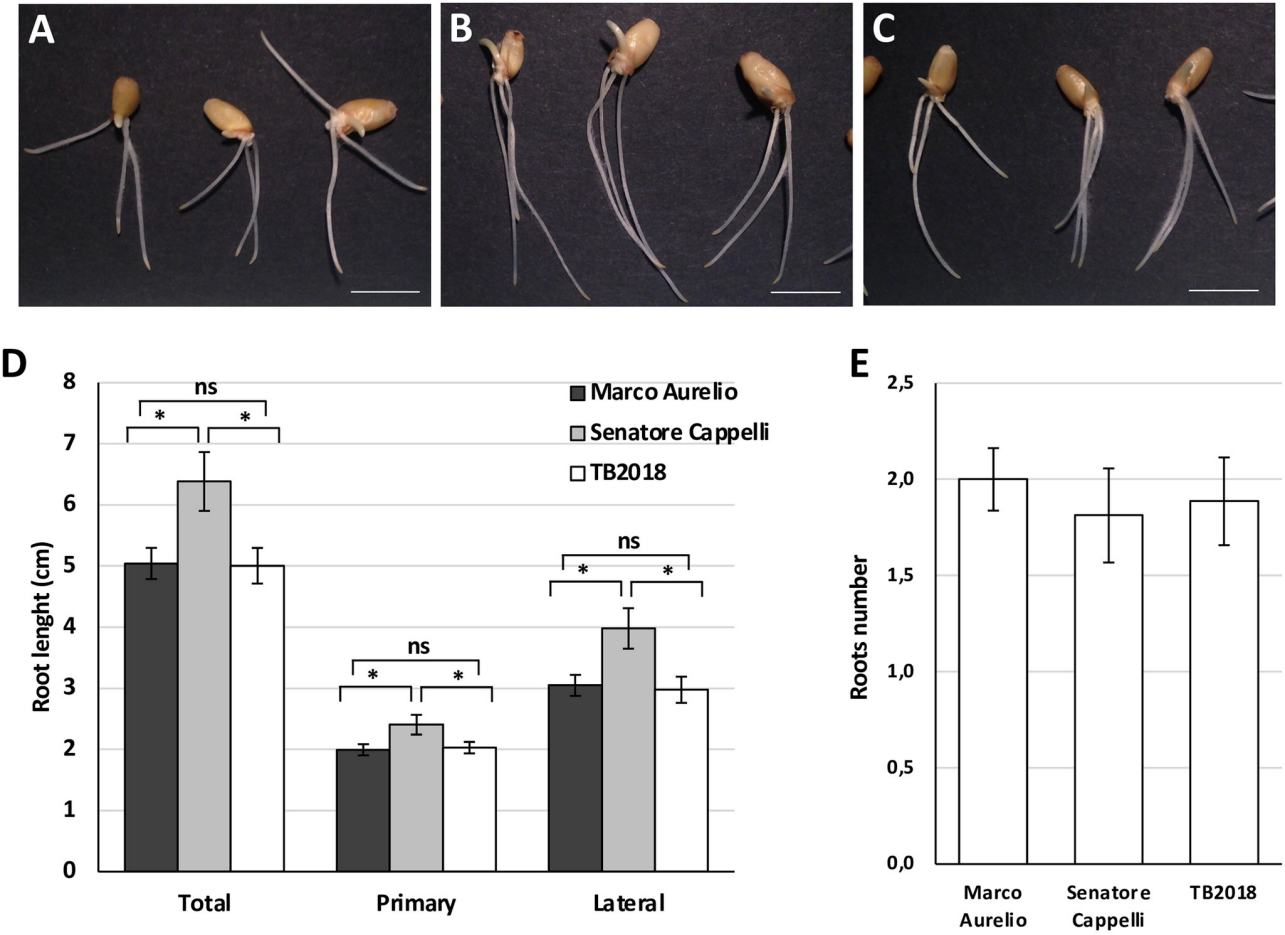
Differences in root length at the seedling stage. Representative images of the three wheat genotypes germinated on paper: ‘Marco Aurelio’ (A), ‘Senatore Cappelli’ (B) ‘TB2018’(C). Plants were grown *in vitro* on paper and parameters were measured at 3 days after sowing (DAS). Length of primary root, lateral root, and total roots (D) as well as number of lateral roots (E) were measured for each plant. Values are means of 32 independent biological replicates. Asterisks indicate statistical significance using Student’s t test (*P < 0.05, ns = not significant). Error bar = standard error.

Differences in root development were instead observed (Fig 3A–3C).

A reduction in root elongation was detected for ‘TB2018’ if compared to ‘Senatore Cappelli’. The reduction was found for the primary root length as well as for the sum of primary and lateral roots lengths measured at the time of sampling (Fig 3D). However, root length of ‘TB2018’ was like that observed for ‘Marco Aurelio’ and the total number of lateral roots was similar among the three accessions (Fig 3E).

### Genome-wide characterization of the ‘TB2018’ landrace

Overall, data on protein profile and plant development provided evidence supporting the hypothesis that the ‘TB2018’ landrace and the registered ‘Senatore Cappelli’ *durum* wheat cultivars are somehow related. It also highlighted the presence in ‘TB2018’ of distinct traits of agronomic importance. Therefore, to gain insight into the relationship between TB2018 and other wheat genotypes and in view of its future exploitation as a source of interesting genetic variants, for the ‘TB2018’ landrace a low-coverage resequencing of its genome was performed and data were compared against the available ‘Svevo’ reference genome [13]. The bioinformatic pipeline comprised a first quality control of reads to discard those that did not pass a minimum threshold value [29]. The remaining reads were mapped against the ‘Svevo’ genome and yielded the Variant Call Format (VCF) files used to feed the Variant Effect Predictor (VEP) software. Generated data were analyzed at the single chromosome level yielding 14 sets of data corresponding to the 14 chromosomes of the reference genome covering the 7 A-series and 7 B-series haploids (S1–S14 Files).

The number of processed variants per chromosome varied from 95,986 (chromosome 1A) to 437,679 (chromosome 2B) with a range of putatively affected genes between 390 (chromosome 4B) and 971 (chromosome 2B) (S2 Fig, S3 Table). Interestingly, all the chromosomes of the B series featured a higher number of variants than their equivalents of the A series, with a notable 4.5-fold difference between pair 1B and 1A.

Besides several expected low-quality variants that were discarded from the analysis, most variants detected fall in the large intergenic non-coding regions and their information value is expected to be negligible in comparison to that of coding genes. The results of the chromosome-wide analysis are shown in S3 Fig.

Several of the hundreds of genes putatively affected by variants possess Gene Ontology descriptors that are linked to the morphological traits that distinguish ’TB2018’ from ’Senatore Cappelli’ (S4 Fig., S4 Table).

The most represented descriptor in the Gene Ontology “Biological process” domain is “Defense response” (GO:0006952), followed by “Protein phosphorylation” (GO:0006468). The fifth position is occupied by “Regulation of DNA-templated transcription” (GO:0006355) which includes transcription factors.

In particular, the analysis detected several variants in genes and gene families associated with known QTL loci for various physiological and agronomic traits. A special case is the occurrence of a mutation (a stop gained at protein position 438: Y/*) affecting a low-molecular-weight (LMW) glutenin subunit gene (TRITD1Av1G002790), featuring 4 transcripts (splice variants), 16 orthologues and 5 paralogues in the ‘Svevo’ reference genome.

The search for variants in regulatory genes involved in root development has highlighted a mutation (a stop gained at protein position 88: Q/*) in a member (TRITD1Av1G207410) of the WRKY family, one of the largest TF families in plants whose members, in addition to stress response and defense regulation, significantly determine plant development and growth [57].

Transposable elements (TEs) are distinct genetic elements that move and spread within the host genomes in multiple copies. TEs can be major constituents in plant genomes and can drive genome evolution, plasticity, and expansion [58, 59].

The analysis of repetitive elements carried out on the ‘TB2018’ genome by RepeatMasker [42] revealed 9,778,301 features distributed along the 14 chromosomes with a maximum of 838,038 on chromosome 3B and a minimum of 558,432 on chromosome 1A (S5a Fig). An example of the formatted output of the GenSAS server is shown (S5b Fig). Moreover, a study of the chromosome-wide distribution of the three main long terminal repeat (LTR) retrotransposon superfamilies *Gypsy*, *Copia* and *EnSpm* has been carried out (S5c Fig). The complete dataset is accessible at https://doi.org/10.13130/RD_UNIMI/SLKJLY.

To investigate the origin of ‘TB2018’, a genome-wide comparison was run against a dataset of SNP genotypes covering 3,541 marker loci of 259 durum wheat accessions released since the early 1900s and representative of landraces, old cultivars, and modern varieties [45].

The full list of the 3,541 used SNP loci and corresponding genotypes is shown in the accompanying file (S15 File). The vast majority (99.10% of total) of the SNP loci were properly called, while only a small number, 32 SNP loci (0.90%) were not called by the analytical pipeline.

Notably, two groups of such loci fell in contiguous portions of the genome spanning positions 11 Mb to 18.5 Mb and 412 Mb to 420 Mb of chromosome 1A and chromosome 5A, respectively. The unrepresented regions might be due to insufficient coverage of the corresponding genomic sequences at low-level (5x) sequencing, although, due to their extension and contiguity, structural variations (SVs) are a more probable alternative explanation. Interestingly, SVs caused by tandem repeats occurring at chromosome 5A have already been recognized as a factor affecting recombination and major cytological gene-affecting rearrangements in wheat [60]. Further investigation of the nature of these regions will be addressed in future work.

**Fig 4:**
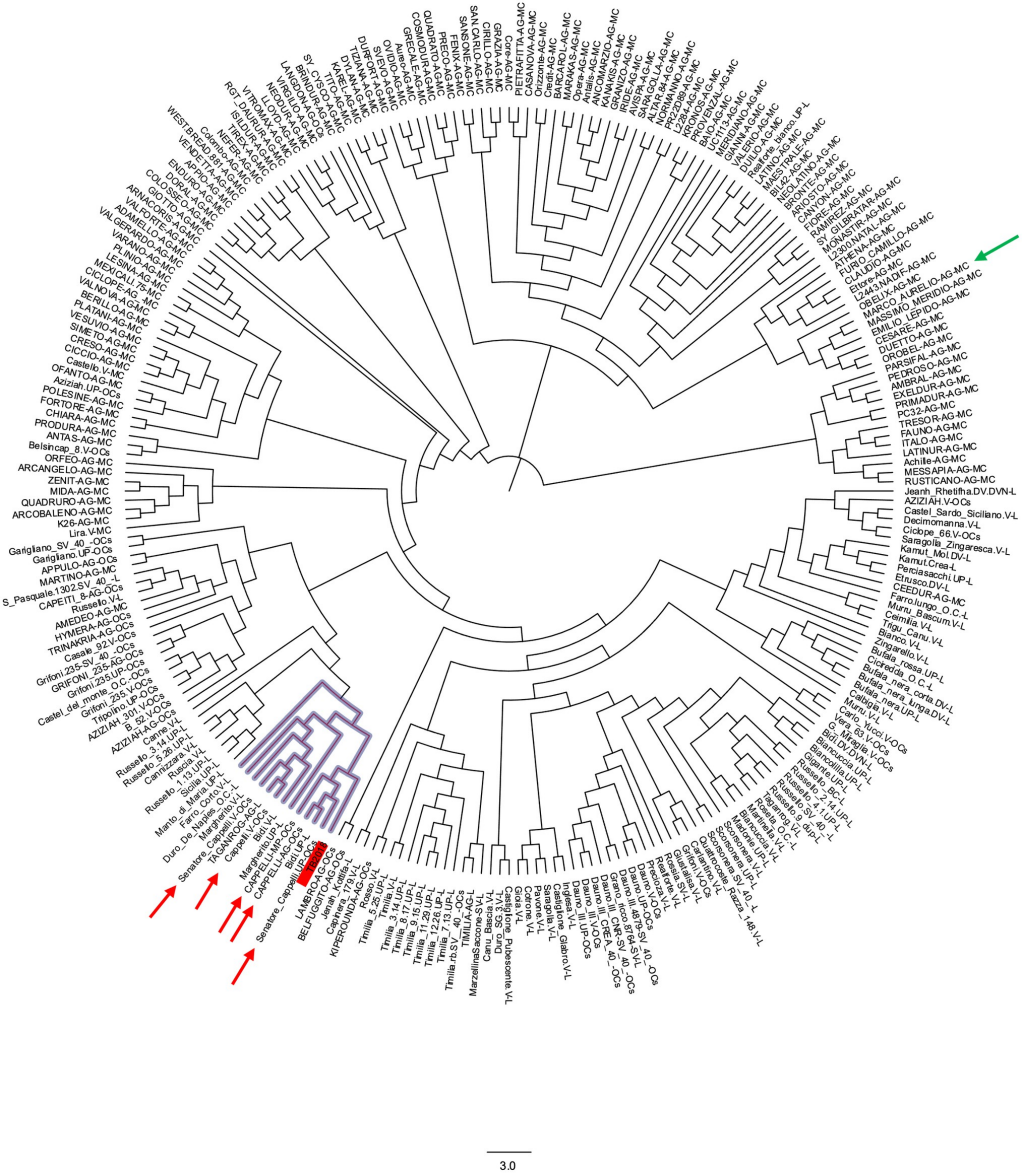
Cluster analysis of the 259 durum wheat accessions compared to ‘TB2018’. The ‘Cappelli’ accessions are indicated by red arrows; ‘Marco Aurelio’ is indicated by a green arrow; ‘TB2018’ is included in a red box. The subcluster including the ‘Cappelli’-like accessions is highlighted in blue.

The reconstructed phylogenetic tree placed ‘TB2018’ in proximity with a group of accessions sharing the designation ‘Senatore Cappelli’, among which ‘Senatore Cappelli V-OCs’, ‘Cappelli V-OCs’, ‘Cappelli-MP-OCs’, ‘Cappelli AG-OCs’, and ‘Senatore Cappelli UP-OCs’. Moreover, in the same group are also present ‘Margherito.UP-L’ and ‘Bidi.UP-L’ which are known to be close relatives of ‘Senatore Cappelli’ (Fig 4).

The genome-based comparison of ‘TB2018’ to a large set of wheat germplasm resulted in agreement with data coming from the morpho-physiological characterization that placed ‘TB2018’ close to the ‘Cappelli’ reference (Fig 1).

To further investigate the placement of ‘TB2018’ among the ‘Cappelli’ accessions described in [45] we performed an analysis with the G-DIRT software [54], a tool for the identification and removal of duplicate germplasm based on identity-by-state analysis using SNP data. The results indicate, out of the 3,509 SNP loci left from the genotyping test described above, that 613 markers were retained after linkage disequilibrium (LD) pruning, and 511 markers were retained after applying the Hardy-Weinberg Equilibrium (HWE) data filtration threshold of 0.05 and Heterozygosity threshold of 0.1. All the six input genotypes were retained after missing data filtration of 10%. Four genotypes were retained after removing duplicates with less than 0.1% of Homozygous difference (S3 Text).

## Discussion

Landraces are considered an attractive breeding option since they are a reservoir of interesting traits and genetic biodiversity of wheat landraces has always captured the attention of researchers.

For example, storage proteins, of which the composition and relative abundance are known to be affected by various environmental factors and growing practices, represent a major target for breeders and food technologists [61, 62]. The search for new alleles controlling seed proteins in the germplasm of durum wheat testifies the interest in this field and the potential for the food industry [63]. In addition, Mediterranean *durum* wheat landraces have previously shown high levels of polymorphism in glutenin subunit composition and its relationship with gluten strength, proving to be valuable sources of diversity for quality traits [64]. The differences observed in HMW glutenins could potentially have implications from a technological point of view since this class of storage proteins has been reported to play an important role for the bread making process. In particular, they are relevant determinants of gluten elasticity and functionality, despite being minor protein components [65].

‘TB2018’ represents a new landrace of *Triticum durum* which was first identified as naturally occurring in a specific environment in the South of Italy and selected for its reduced height. The physical distance of the site of its discovery from cultivated fields posed a question as to its origin and nature, although morphological analysis seemed to place ‘TB2018’ close to the ‘Senatore Cappelli’ cultivar. Following the detailed characterization conducted in this work with the traditional agronomical and laboratory investigation techniques, its similarity with ‘Senatore Cappelli’ has been confirmed, but also a distinctive trait, i.e., the reduced plant elongation in comparison with ‘Senatore Cappelli’ and other wheat landraces, has been highlighted (Fig 1). In addition, differences in seedling root development were detected for ‘TB2018’ when seeds were germinated in controlled conditions: a reduction in root elongation was observed in the early stages of seedling growth if compared with the reference cv. ‘Senatore Cappelli’.

One of the most interesting aspects of this work consists in the re-sequencing, to the best of our knowledge, of the first durum wheat landrace. We have adopted (and described) a novel approach, which constitutes the first example of comparison of whole-genome resequencing (WGR) data to genotyping data obtained through a commercial chip-based technology [40]. While the minimum number of individuals that need to be genotyped in a chip-based platform (usually 96), is normally a limiting factor for the analytical costs, we demonstrate that it is possible to compare even a single sample resequenced at low coverage (5x) with data previously obtained through chip-based technology. Data obtained allowed us to explore both the genetic biodiversity and the origin for this accession. The analysis placed the landrace in the group of ‘Senatore Cappelli’ cultivars. The many genomic variations encountered in this landrace, affecting, to a variable degree of importance, several genes recognized as regulators of various biochemical pathways, might account for the major differences observed until now through the comparison of ‘TB2018’ with the reference ‘Senatore Cappelli’ and other known cultivars. To further contribute to the characterization of durum wheat genome structure, we report the distribution of the repetitive elements belonging to the major retrotransposon classes. That the repetitive nature of the wheat genome is mainly the result of a high content of transposable elements and their role in genome plasticity, evolution and gene regulation is widely recognized [66, 67].

Despite the challenging genome size (ca. 10 Gigabases) that makes the analysis of this species of uncommon complexity, the possibility of predicting the impact of genomic variation on physiological characteristics will open the way to a rational characterization and exploitation of naturally occurring variation.

In this context, one example is constituted by the detection of a mutation in a regulatory gene belonging to the WRKY family, which represents a candidate gene responsible for the variation observed in plant or root elongation. The product of TaWRKY51, another member of this family, was shown to increase lateral root formation through the regulation of ethylene biosynthesis in wheat. The same study also reported that TaWRKY51 regulates lateral root formation via the ethylene and auxin signaling pathways [68]. More recently, the combined role of TaWRKY51 in positively regulating root architecture and grain yield contributing traits has been assessed [69]. Studies have also shown that the number and length of root hairs are increased in a knockout mutant of AtWRKY75 compared to the WT, suggesting that AtWRKY75 is a negative regulator of root hair development [70]. A study on rice has demonstrated that OsWRKY36 plays a negative role in regulating grain size and plant height through directly binding the promoter of the SLR1 gene and protecting it from GA-mediated degradation [71].

These observations sustain the hypothesis that the mutation possibly disrupting the WRKY TF identified in the present investigation could thus explain the changes in plant height or root structure of ‘TB2018’ in comparison to ‘Senatore Cappelli’.

Genetic diversity of plant size and structure can profoundly affect qualitative and quantitative traits of the production, including seed quality [72–74], influence agronomic practices, yields and even technological applications in the energy industry [75]. In particular, the plant root is a central architectural element that profoundly controls and affects the entire plant physiology and its ability to respond to the environmental conditions and stresses. The ‘steep, cheap, and deep’ root ideotype for improved nitrogen and water uptake is based on morphological, anatomical, and physiological traits that promote rapid exploration of deep soil domains [76]. The availability of candidate genes represents an important tool for the improving of wheat cultivars.

Roots are complex, dynamic organs interacting with the soil where they develop and play a fundamental role in plant-environment interaction. Root phenotypes can substantially improve soil resource capture and CO_2_ sequestration by crop plants. These qualities afford multiple benefits for agroecosystems and the global environment. In high-input agroecosystems, crops with reduced demand for fertilizers and water can reduce production costs and the risk of losses from drought, while also countering adverse environmental impacts due to input use [77]. For wheat cultivars an improved root apparatus is essential for their adaptation to the area of cultivation as well as to fit the plant to unstable environmental conditions. Unlike common wheat (*Triticum aestivum* L.), durum wheat is primarily grown in marginal environments of the Mediterranean and semiarid regions of the world, where moisture is mostly provided through rain. Annual variation in rainfall normally characterizes the Mediterranean environment, with late-season droughts occurring frequently. When droughts coincide with the flowering or grain-filling phase, yield and grain quality can be dramatically affected. Furthermore, due to the warming climate, the steady reduction of seasonal rainfall in the Mediterranean region is predicted to adversely affect the cultivation of durum wheat [77]. According to modeling studies, wheat yield can increase by 55 kg ha^−1^ on average for each millimeter of water extracted from the soil after anthesis [78–80].

Besides crop environmental adaptation, from the agronomic point of view, chemical input reduction in agricultural systems is being strongly demanded with the aim to improve the quality and the safety of food/feed products in an environmentally sustainable perspective [81]. In this respect, we have evidence that the seed protein content (SPC) of this landrace is not significantly affected under a partial reduction of total nitrogen input. A possible explanation for this behavior can be found in the alleles that reduce plant size and that might mitigate the overall biomass reduction.

Overall, ‘TB2018’ appears promising as a possible candidate for both direct adoption in the cultivation and for its use as a donor of interesting alleles for breeding.

A more detailed description of ‘TB2018’ should be undertaken following the huge amount of information provided by the exploration of its genomic diversity which has been only partially addressed in this work. In fact, the exploitation of naturally occurring genetic resources has been recognized as an invaluable, yet traditional way to address breeding [6, 82, 83]. The close interplay between genetics, environment and cultivation practices is at the basis of modeling this species for enlarging its cultivation area worldwide [84]. For example, nitrogen increases leaf greenness which, in turn, is expressed by chlorophyll content, plant vitality, increase in fresh biomass and yield formation in the generative growth stages of durum wheat [85, 86]. However, higher nitrogen rates can decrease plant resistance to lodging [87]. The model developed by Berry and Spink [88] predicted that severe lodging up to 90° from the vertical plane can reduce yields by approximately 61%.

We expect that the further characterization of this landrace at the molecular, chemical, and agronomical level will generate additional evidence of the physiological basis of the traits that have attracted our attention. The present work is also a tribute to the ’Senatore Cappelli’ variety which has been cultivated for decades and has contributed to the growth of cereal growing and the Italian agri-food industry. It has also led the way to the modern genetic improvement of durum wheat and still today feeds a supply chain dedicated to the development of high-quality sustainable products.

## Supporting information

S1 Fig Comparison of ‘TB2018 and ‘Senatore Cappelli’. Plants of (A) ‘Senatore Cappelli’ and (B) ‘TB2018’ at the same growth stage in the field are shown.
(PDF)

S2 Fig Distribution of variants detected by genome resequencing of ‘TB2018’ following comparison with the reference durum wheat cv. ‘Svevo’. a) number of processed variants per chromosome of the ‘TB2018’ landrace; b) number of putatively overlapped genes following the Variant Effect Predictor (VEP) analysis.
(PDF)

S3 Fig Distribution of variants per ‘TB2018’ chromosome after comparison with the ‘Svevo’ reference durum wheat genome. (PDF)

S4 Fig Distribution of Gene Ontology (GO) descriptors. (PDF)

S5 Fig Analysis of repetitive elements along the ‘TB2018’ genome. (a) distribution of feature elements per chromosome; (b) a screenshot showing the alignment of repetitive elements along the ‘TB2018’ genome produced by RepeatMasker and displayed on the GenSAS server; (c) distribution of the three main retrotransposon LTR superfamilies *Gypsy*, *Copia*, and *EnSpm*.
(PDF)

S1 File Variant Effect Predictor (VEP) output file for chromosome 1A. (ZIP)

S2 File Variant Effect Predictor (VEP) output file for chromosome 1B. (ZIP)

S3 File Variant Effect Predictor (VEP) output file for chromosome 2A. (ZIP)

S4 File Variant Effect Predictor (VEP) output file for chromosome 2B. (ZIP)

S5 File Variant Effect Predictor (VEP) output file for chromosome 3A. (ZIP)

S6 File Variant Effect Predictor (VEP) output file for chromosome 3B. (ZIP)

S7 File Variant Effect Predictor (VEP) output file for chromosome 4A. (ZIP)

S8 File Variant Effect Predictor (VEP) output file for chromosome 4B. (ZIP)

S9 File Variant Effect Predictor (VEP) output file for chromosome 5A. (ZIP)

S10 File Variant Effect Predictor (VEP) output file for chromosome 5B. (ZIP)

S11 File Variant Effect Predictor (VEP) output file for chromosome 6A. (ZIP)

S12 File Variant Effect Predictor (VEP) output file for chromosome 6B. (ZIP)

S13 File Variant Effect Predictor (VEP) output file for chromosome 7A. (ZIP)

S14 File Variant Effect Predictor (VEP) output file for chromosome 7B. (ZIP)

S15 File List of the 3,541 used SNP loci and corresponding genotypes. (XLSX)

S1 Table List of Italian durum wheat cultivars used in the morpho-physiological characterization. (CSV)

S2 Table List of parameters set for the Variant Effect Predictor (VEP) analysis. (DOCX)

S3 Table Number of variants per ‘TB2018’ chromosome along with their features. (CSV)

S4 Table List of genes putatively affected by severe mutations according to the VEP analysis with the corresponding Gene Ontology descriptors. (XLSX)

S1 Text Details of the bioinformatic pipeline. (DOCX)

S2 Text List of loci and genotypes used for the G-DIRT analysis. (TXT)

S3 Text Parameters and output of the G-DIRT analysis. (DOCX)

S1 Raw images Raw image version of Fig 2. (PDF)
