# Supplementary figures and images for: Whole-genome resequencing-based characterization of a durum wheat landrace showing similarity to ‘Senatore Cappelli’
Source: PLoS One. 2023 Sep 21;18(9):e0291430. doi: 10.1371/journal.pone.0291430 (PMC10513328; doi:10.1371/journal.pone.0291430)

S1 Fig

A

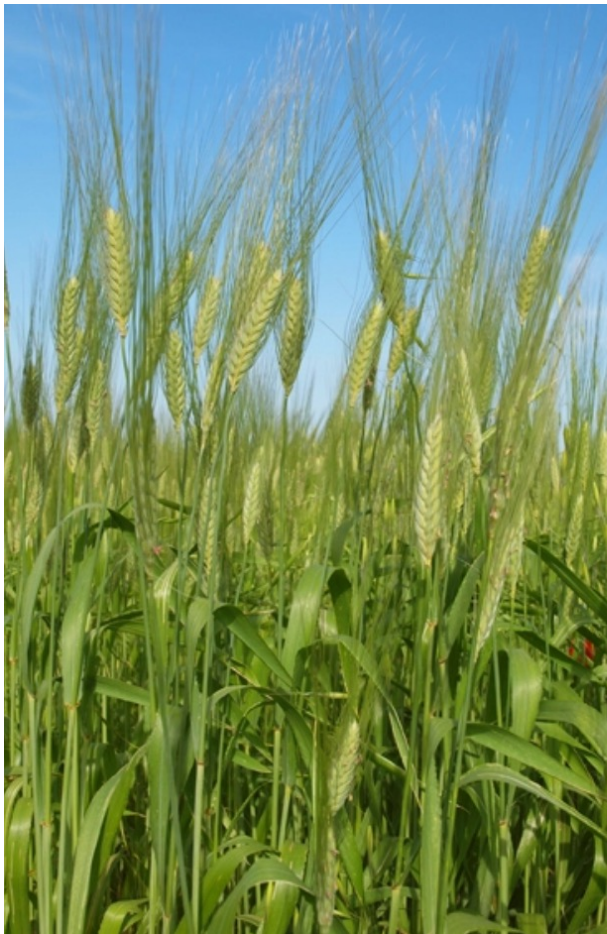

B

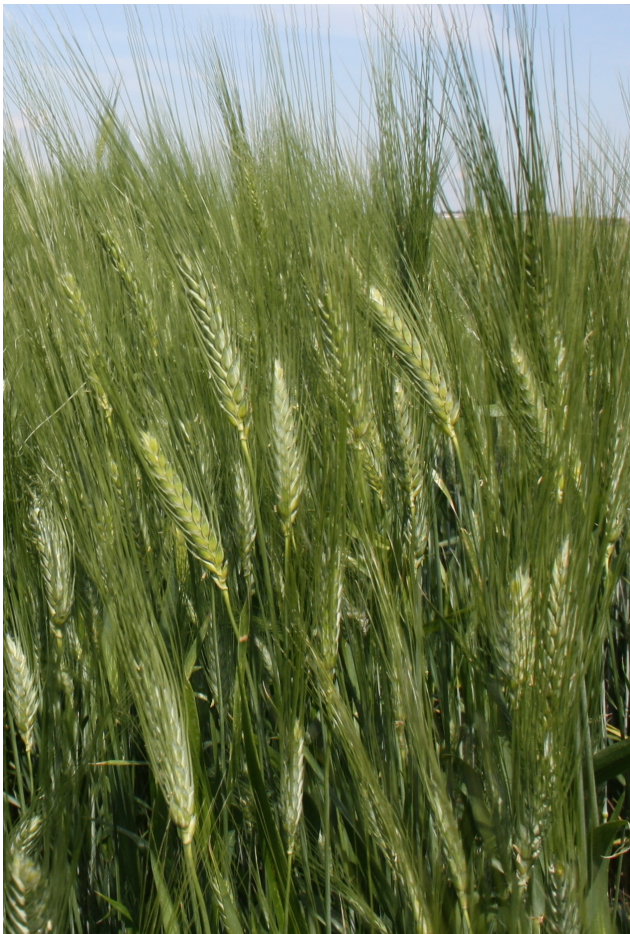

Supplement: S1 Fig — Plants of (A) ‘Senatore Cappelli’ and (B) ‘TB2018’ at the same growth stage in the field are shown. (PDF) [file pone.0291430.s001.pdf]

S2 Fig

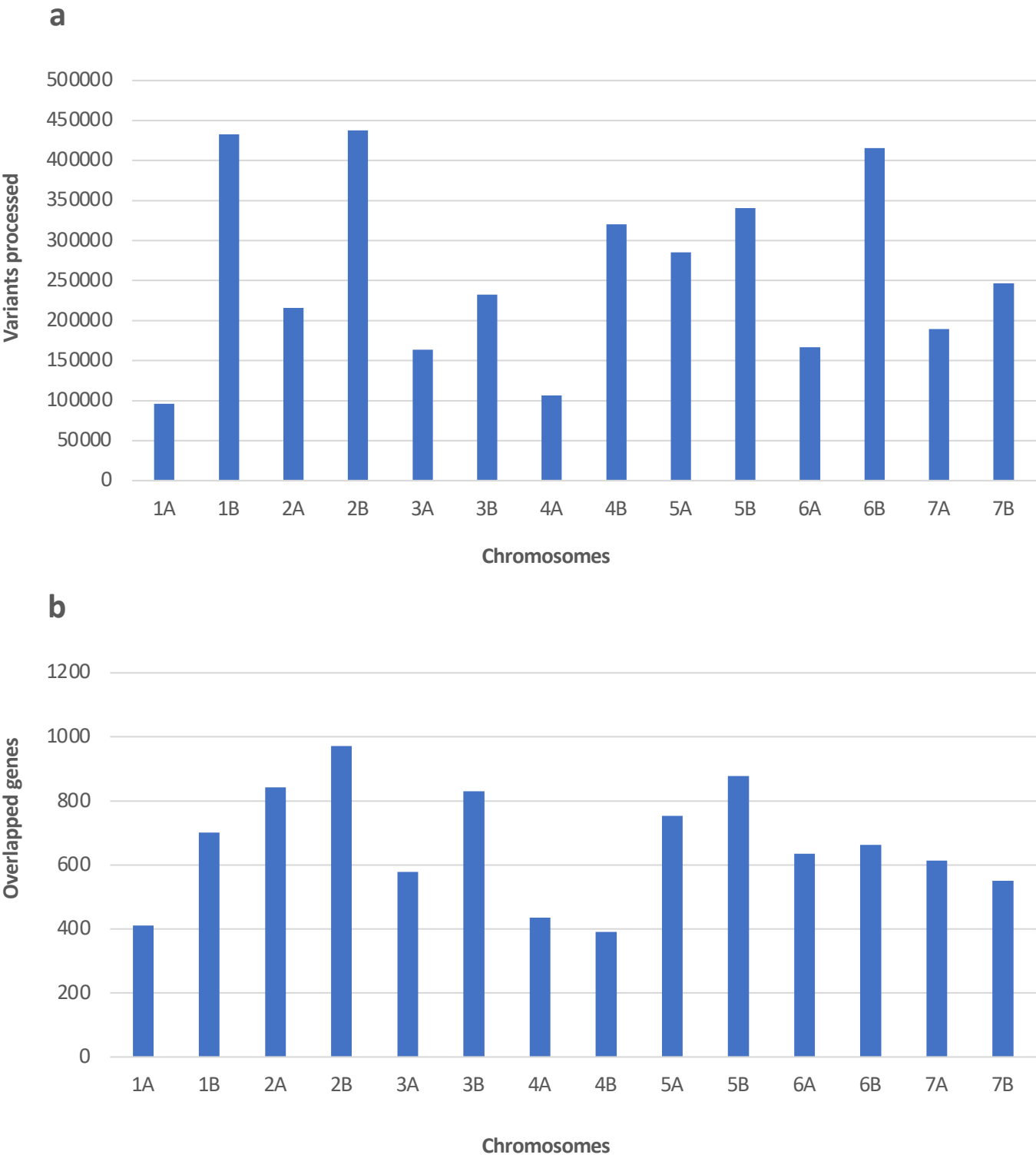

Supplement: S2 Fig — a) number of processed variants per chromosome of the ‘TB2018’ landrace; b) number of putatively overlapped genes following the Variant Effect Predictor (VEP) analysis. (PDF) [file pone.0291430.s002.pdf]

S3 Fig

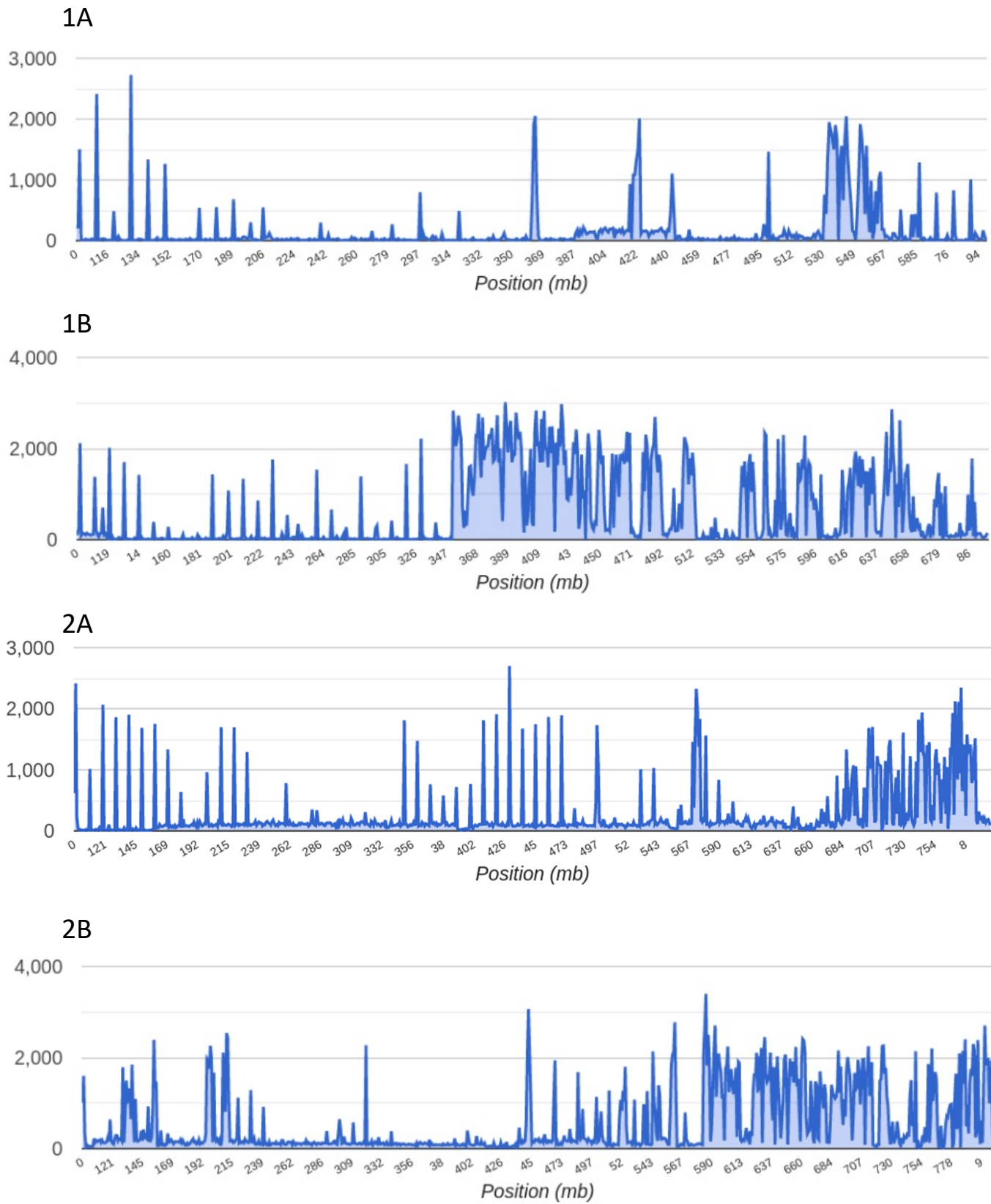

S3 Fig (continued)

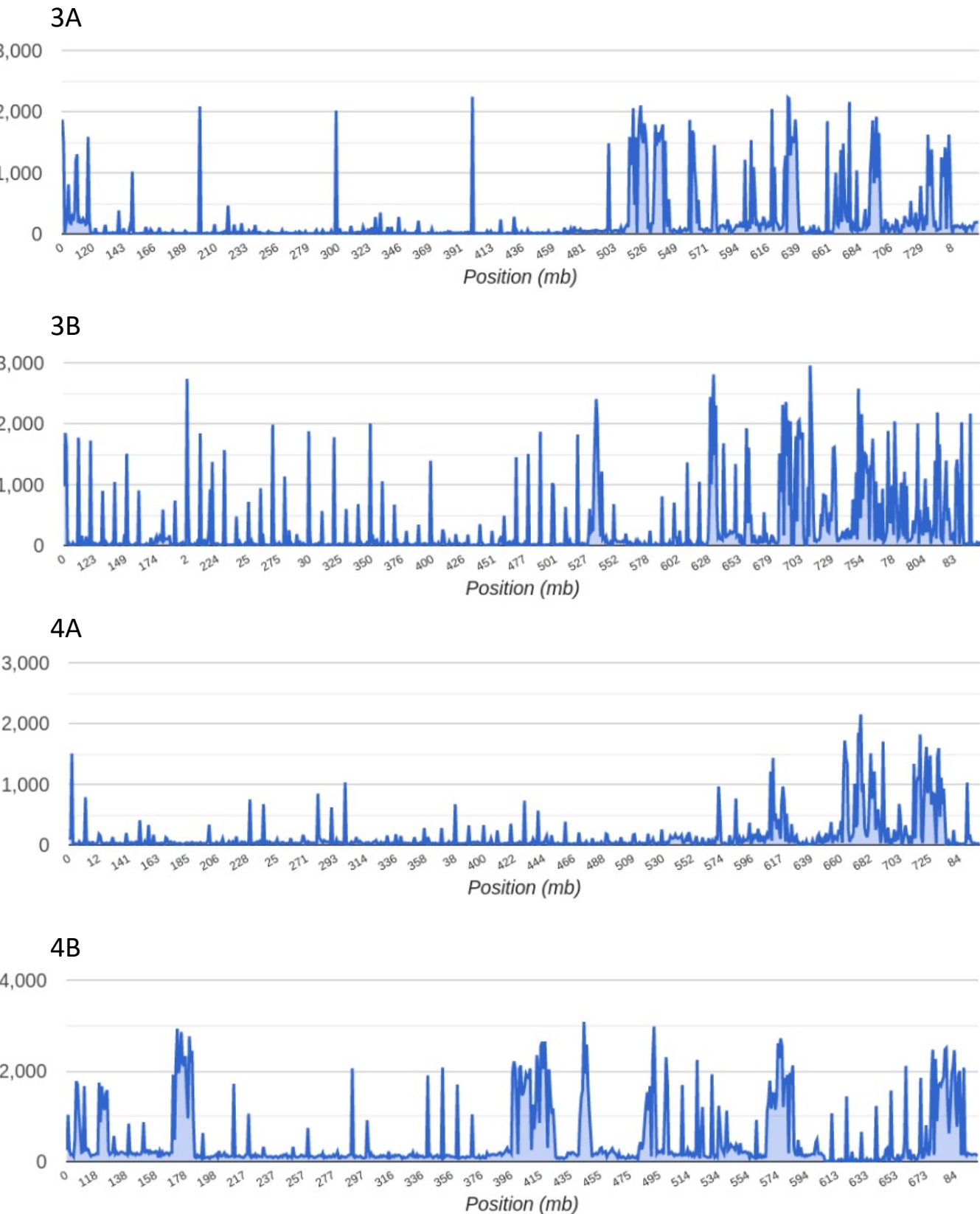

S3 Fig (continued)

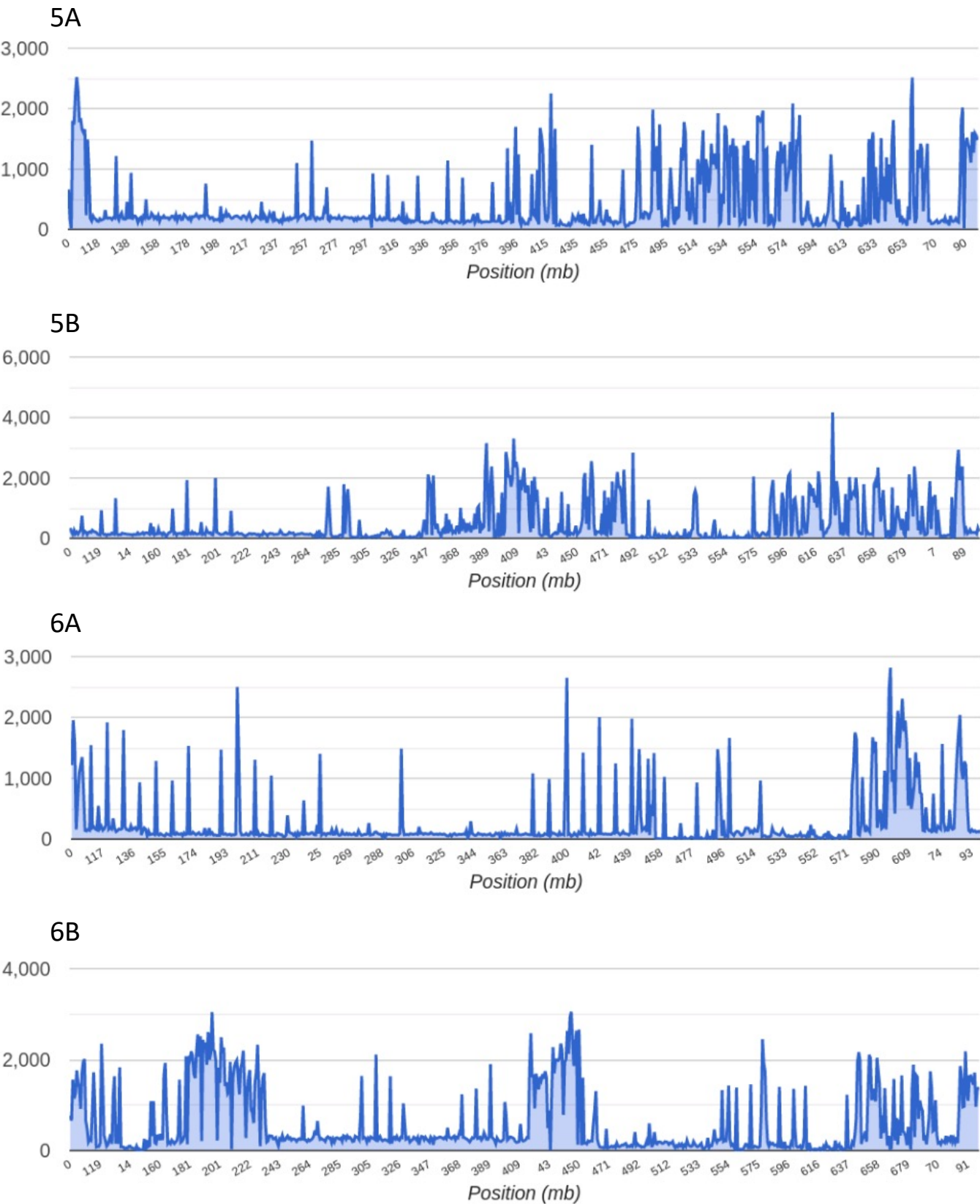

S3 Fig (continued)

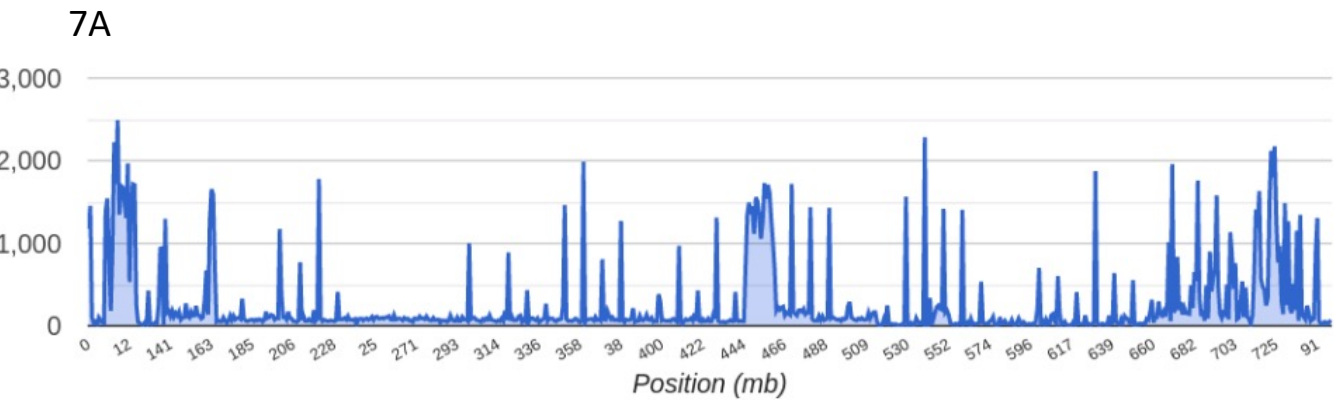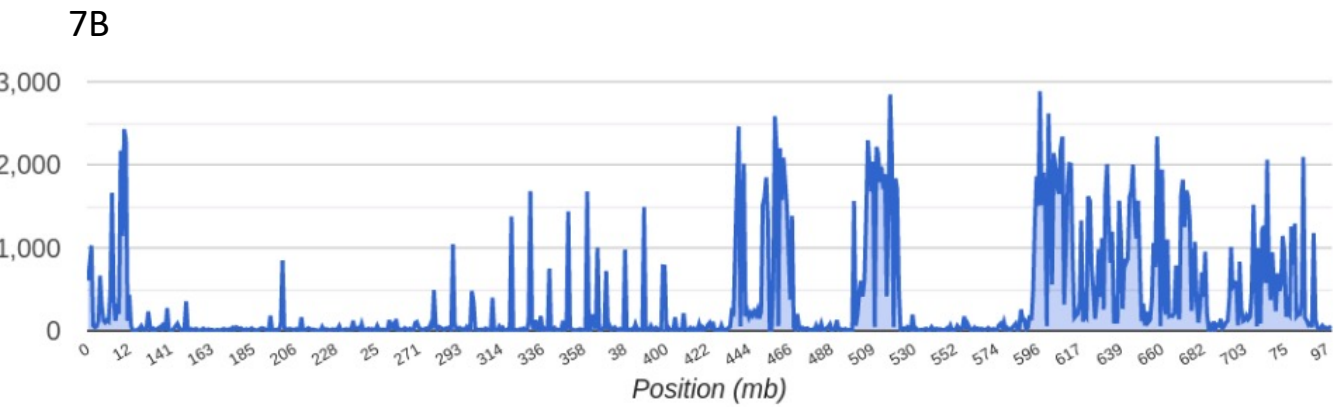

Supplement: S3 Fig — (PDF) [file pone.0291430.s003.pdf]

**S4 Fig**

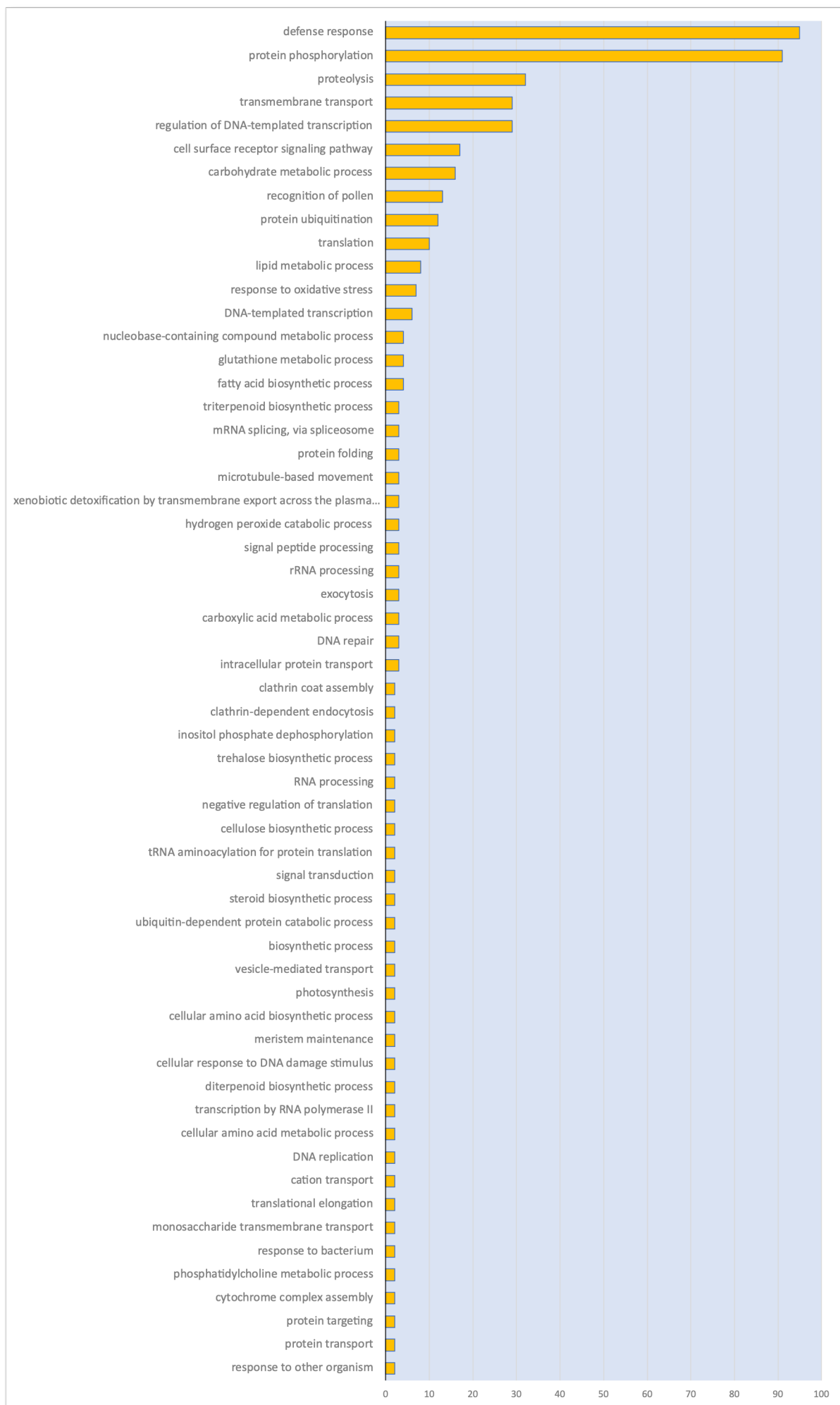

## S4 Fig (continued)

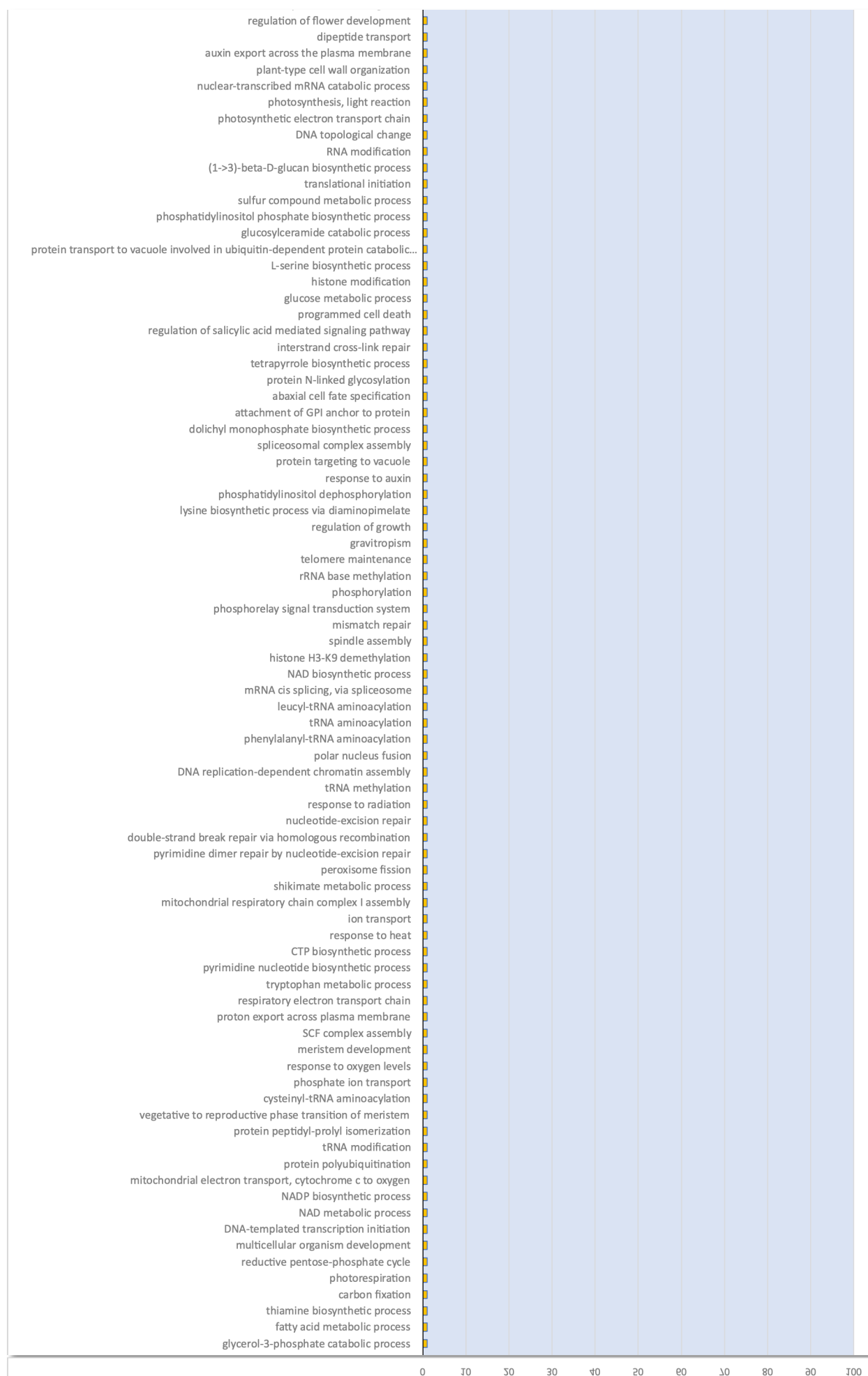

S4 Fig (continued)

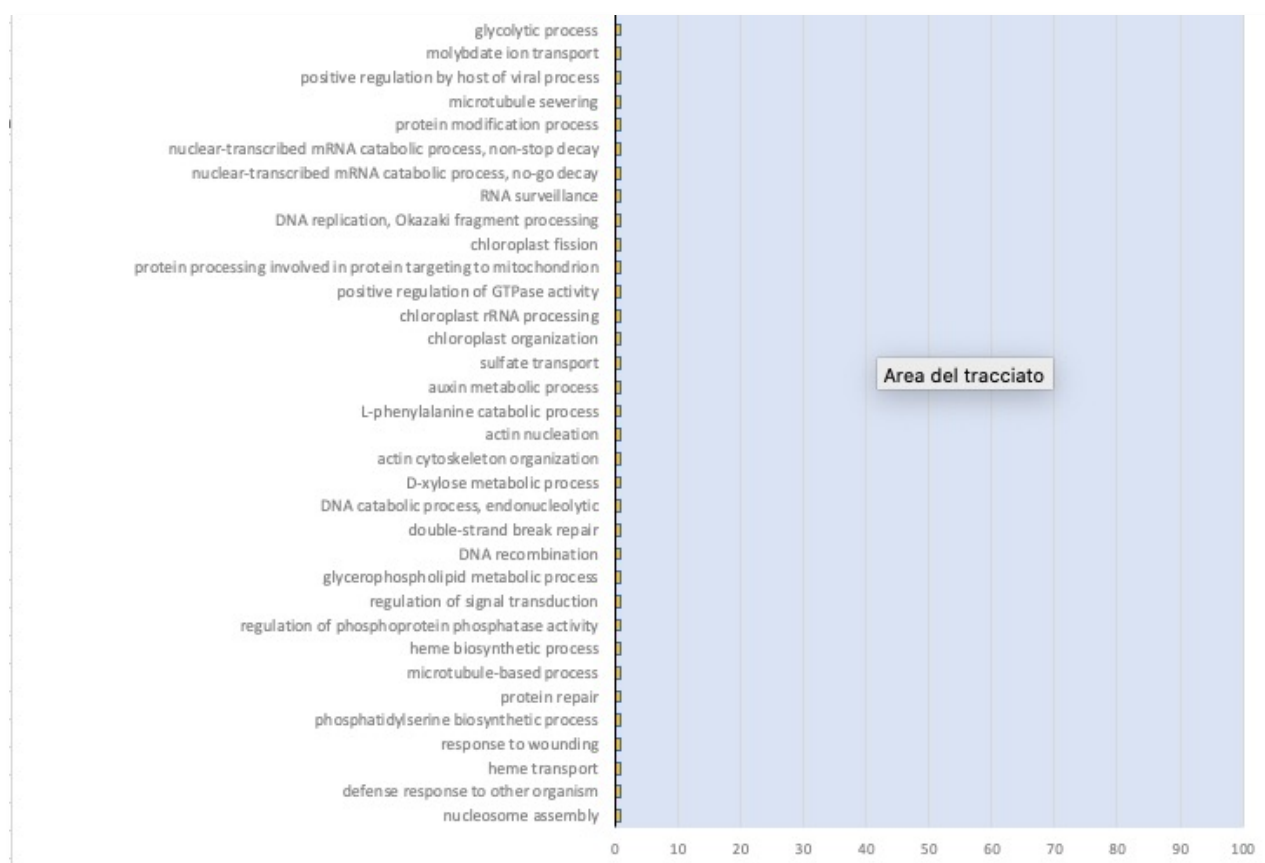

Supplement: S4 Fig — (PDF) [file pone.0291430.s004.pdf]

S5 Fig

A

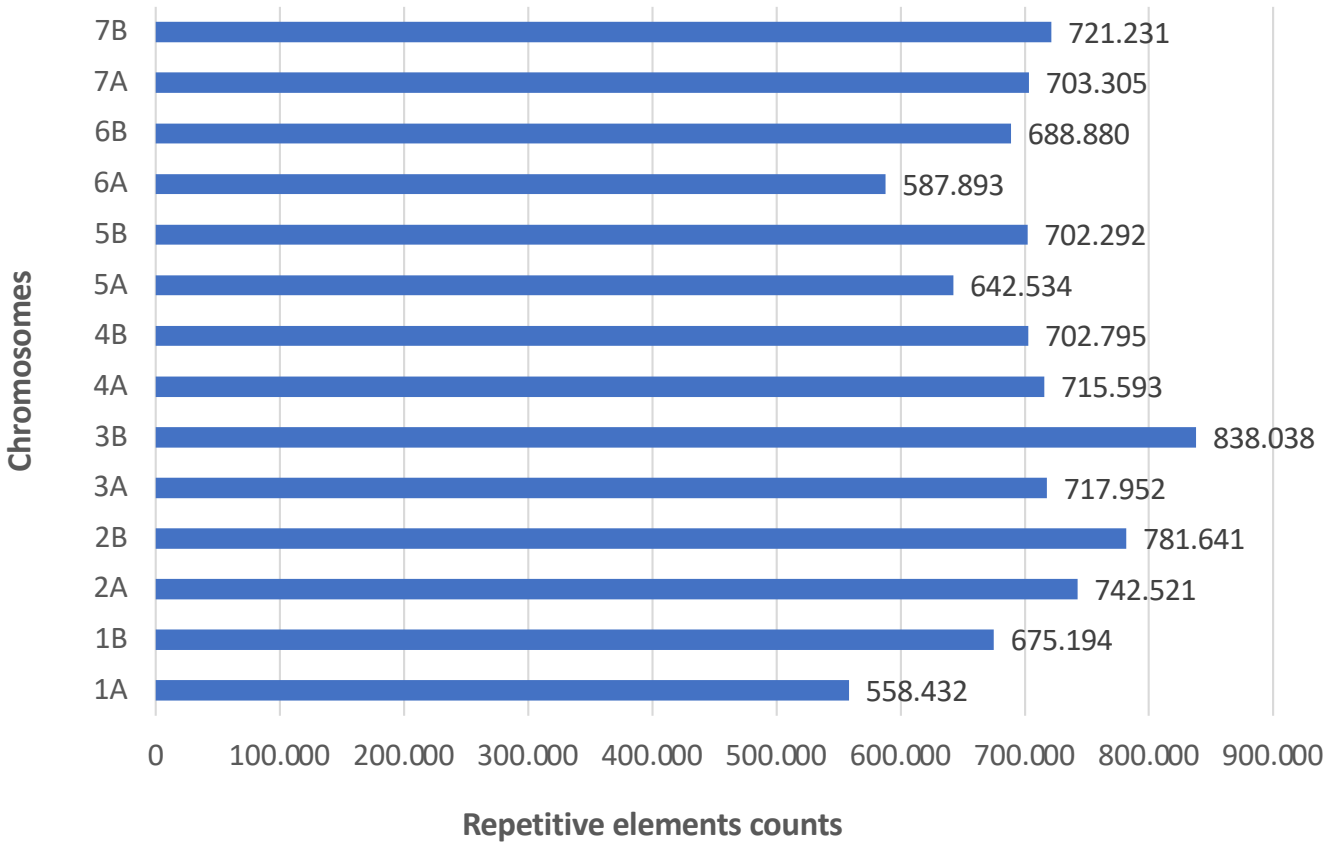

B

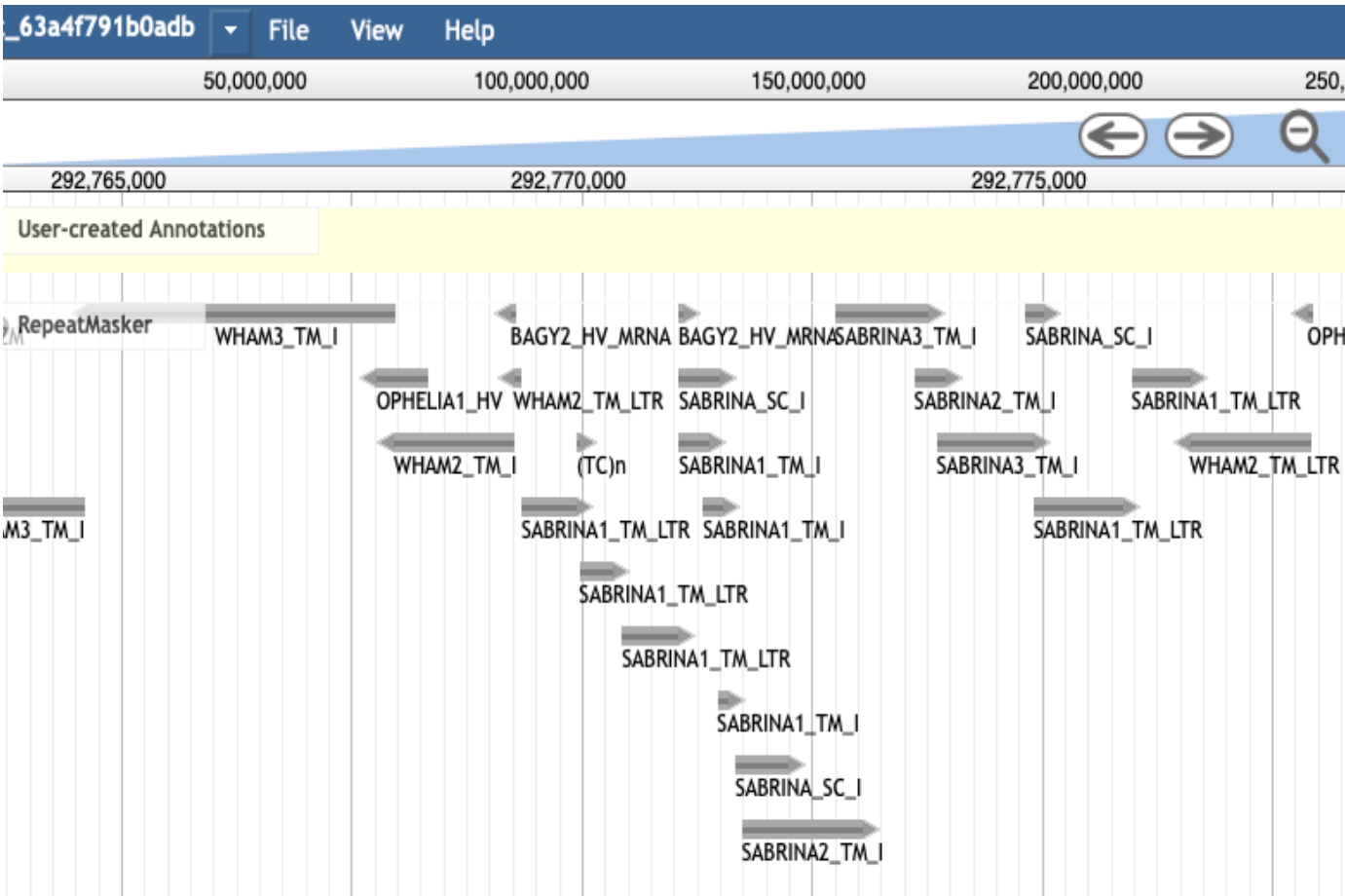

S5 Fig (continued)

C

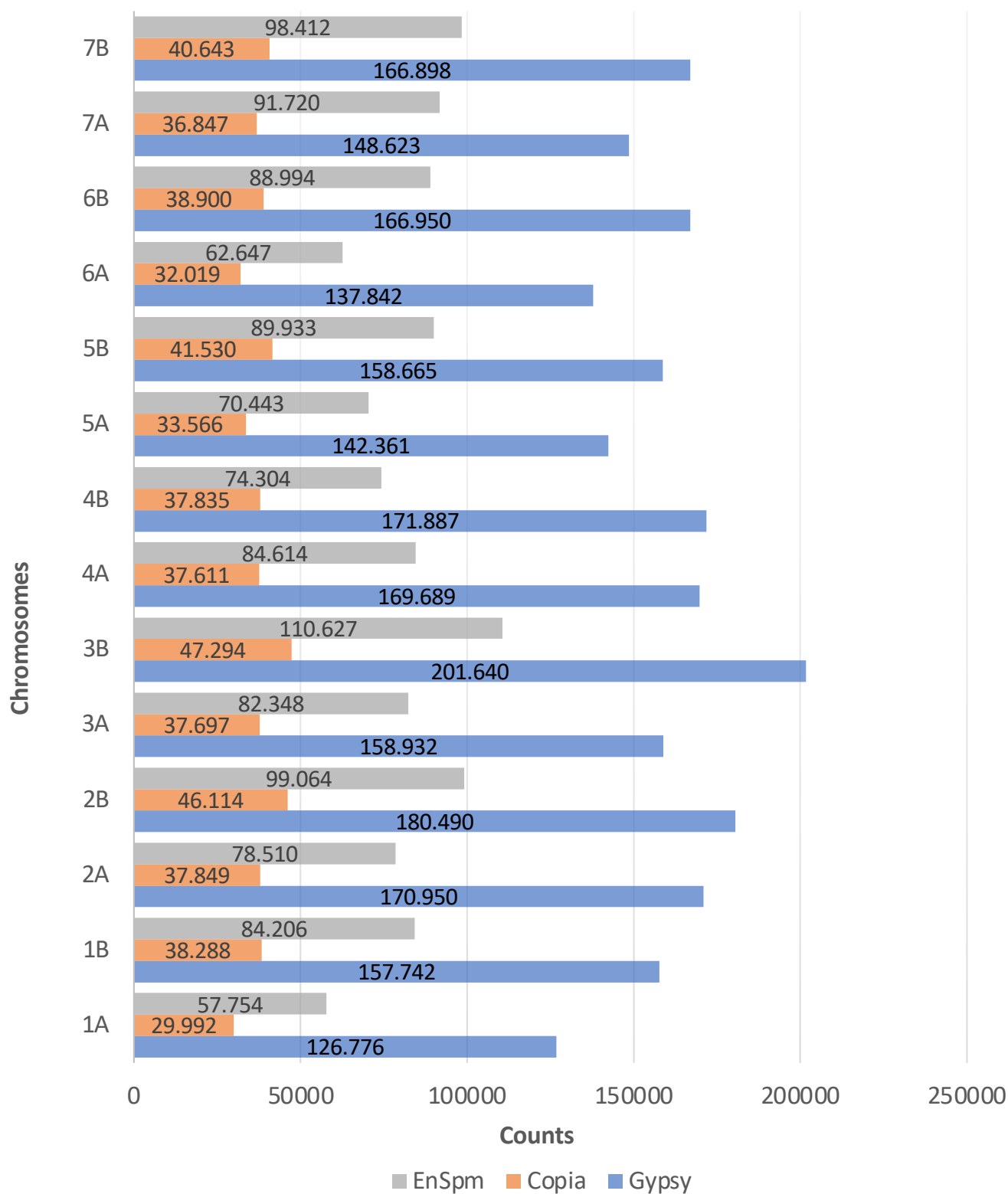

Supplement: S5 Fig — (a) distribution of feature elements per chromosome; (b) a screenshot showing the alignment of repetitive elements along the ‘TB2018’ genome produced by RepeatMasker and displayed on the GenSAS server; (c) distribution of the three main retrotransposon LTR superfamilies Gypsy, Copia, and EnSpm. (PDF) [file pone.0291430.s005.pdf]

MW

X

X

X

A

B

C

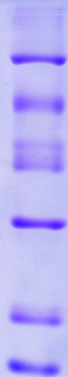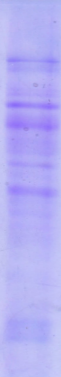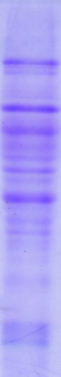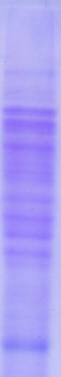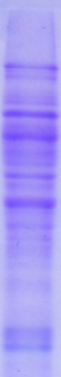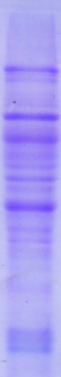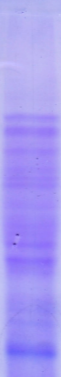

Supplement: S1 Raw images — (PDF) [file pone.0291430.s028.pdf]
