# Supplementary material for: Whole-genome resequencing-based characterization of a durum wheat landrace showing similarity to ‘Senatore Cappelli’
Source: PLoS One. 2023 Sep 21;18(9):e0291430. doi: 10.1371/journal.pone.0291430 (PMC10513328; doi:10.1371/journal.pone.0291430)
Supplement: S2 Table — (DOCX) [file pone.0291430.s022.docx]

**S2 Table**. List of Variant Effect Predictor (VEP) parameters/values.

| **Parameter^a^** | **Value** |
| --- | --- |
| Buffer size | 5000 |
| Exon and intron numbers | Enabled |
| Find co-located known variants | Enabled |
| Gene symbol | Enabled |
| Protein | Enabled |
| Protein domains | Enabled |
| Restrict results | Disabled |
| Return results for variants in coding regions only | Enabled |
| Right align variants prior to consequence calculation | Disabled |
| SIFT | Prediction and score |
| Transcript biotype | Enabled |
| Transcript version | Enabled |
| UniProt | Enabled |
| Upstream/Downstream distance (bp) | 5000 |

**^a^** See [30] for a description of parameters
