# Supplementary material for: Whole-genome resequencing-based characterization of a durum wheat landrace showing similarity to ‘Senatore Cappelli’
Source: PLoS One. 2023 Sep 21;18(9):e0291430. doi: 10.1371/journal.pone.0291430 (PMC10513328; doi:10.1371/journal.pone.0291430)
Supplement: S1 Text — (DOCX) [file pone.0291430.s025.docx]

**Details of the bioinformatic pipeline**

- Cutadapt v1.11 was run on raw fastq data using the following parameters:

--anywhere (on both adapter sequences) --overlap 5 --times 2 --minimum-length 35 --mask-adapter

- Mapping of ‘TB2018’ reads against the ‘Svevo’ reference genome was done using the BasePairQualStats and CoverageStats commands with the following parameters:

java -jar NGSEPcore.jar SingleSampleVariantsDetector \

-maxAlnsPerStartPos 2 \

-minQuality 40 \

-maxBaseQS 30 \

-r /home/users/filippo.geuna/genomica/Data_Frumento_duro_Tateo_copy/GCA_900231445.1.fasta \

-i /home/users/filippo.geuna/genomica/Data_Frumento_duro_Tateo_copy/sorted/Td_Tateo.sorted.bam \

-o /home/users/filippo.geuna/genomica/Data_Frumento_duro_Tateo_copy/variants/population_2.vcf

java -jar NGSEPcore.jar BasePairQualStats \

-r /home/users/filippo.geuna/genomica/Data_Frumento_duro_Tateo_copy/GCA_900231445.1.fasta \

-o /home/users/filippo.geuna/genomica/Data_Frumento_duro_Tateo_copy/sorted/Td_Tateo_readpos.stats \

/home/users/filippo.geuna/genomica/Data_Frumento_duro_Tateo_copy/sorted/Td_Tateo.sorted.bam \

>& /home/users/filippo.geuna/genomica/Data_Frumento_duro_Tateo_copy/sorted/Td_Tateo_readpos.log

java -jar NGSEPcore.jar CoverageStats \

-i /home/users/filippo.geuna/genomica/Data_Frumento_duro_Tateo_copy/sorted/Td_Tateo.sorted.bam \

-o /home/users/filippo.geuna/genomica/Data_Frumento_duro_Tateo_copy/sorted/Td_Tateo_coverage.stats \

>& /home/users/filippo.geuna/genomica/Data_Frumento_duro_Tateo_copy/sorted/Td_Tateo_coverage.log

java -jar /home/users/filippo.geuna/picard-2.3.0-0/picard_2.26.3.jar CollectInsertSizeMetrics \

I=/home/users/filippo.geuna/genomica/Data_Frumento_duro_Tateo_copy/sorted/Td_Tateo.sorted.bam \

O=/home/users/filippo.geuna/genomica/Data_Frumento_duro_Tateo_copy/sorted/Td_Tateo_insertLength.stats \

H=/home/users/filippo.geuna/genomica/Data_Frumento_duro_Tateo_copy/sorted/Td_Tateo_insertLength.pdf \

M=0.5

java -jar /home/users/filippo.geuna/picard-2.3.0-0/picard.jar ValidateSamFile \

I=/home/users/filippo.geuna/genomica/Data_Frumento_duro_Tateo_copy/sorted/Td_Tateo.sorted.bam \

MODE=SUMMARY

- The analysis of repetitive elements of ‘TB2018’ was done on the GensSAS server (see Materials and methods section) using the following parameters:

Search Engine: repeatmasker_engine_sel : ncbi;

Speed/Sensitivity: repeatmasker_speed_sel : -q;

DNA Source: repeatmasker_species_sel : grasrep.ref;

Matrix: repeatmasker_matrix : -gccalc

- The BLAST v. 2.13.0+ search against a local database generated from the ‘TB2018’ was done using the following parameters:

blastn -query /path/to/Infinium_probe_set.fasta -db /path/to/TB2018_consensus_genome.fasta \

-evalue 1e-6 -num_threads 16 -max_target_seqs 1 -max_hsps 1 -outfmt 4 \

-out /path/to/output_blastn.txt

-The ‘getfasta’ command of the ‘Bedtools’ v. 2.25.0 software used the following parameters:

bedtools getfasta -fi /path/to/TB2018_consensus_genome.fasta \

-bed /path/to/TB2018_bedtools_marker_list.bed \

-fo /path/to/output_bedtools.fasta -s -name -tab
