# Supplementary material for: Whole-genome resequencing-based characterization of a durum wheat landrace showing similarity to ‘Senatore Cappelli’
Source: PLoS One. 2023 Sep 21;18(9):e0291430. doi: 10.1371/journal.pone.0291430 (PMC10513328; doi:10.1371/journal.pone.0291430)
Supplement: S3 Text — (DOCX) [file pone.0291430.s027.docx]

**G-DIRT software analysis (parameters and output)**

Parameters

Remove Monomorphic SNPs : TRUE

Generate Cluster: Rectangular

Minor Allele Frequency : 0.05

Missing Data (Marker): 0.05

Missing Data (genotype): 0.1

Linkage Disequilibrium Pruning : 0.75

Homozygous Difference: 0.1

Hardy-Weinberg Equilibrium : 0.05

Marker Heterozygosity: 0.1

Summary

The input hapmap file contains 3509 markers for 6 genotypes.

6 genotypes retained after missing data filtration of 10%.

4 genotypes retained after removing duplicates with less than 0.1 % of Homozygous difference.

613 markers retained after LD pruning at a threshold of 0.75.

511 markers retained after data filtration with HWE threshold of 0.05 and Heterozygosity threshold of 0.1.

Duplicates

| ***Genotype-1*** | ***Genotype-2*** | ***Percentage(%) of difference*** |
| --- | --- | --- |
| Senatore_Cappelli.V.OCs | Cappelli.V.OCs | 0 |
| Senatore_Cappelli.UP.OCs | CAPPELLI.AG.OCs | 0 |
